# Clofarabine induces ERK/MSK/CREB activation through inhibiting CD99 on Ewing sarcoma cells

**DOI:** 10.1371/journal.pone.0253170

**Published:** 2021-06-16

**Authors:** Handan Sevim, Haydar Çelik, Levent Düşünceli, Ceyda S. Ceyhan, Anna Molotkova, Kay Nakazawa, Garrett T. Graham, Jeffrey R. Petro, Jeffrey A. Toretsky, Aykut Üren

**Affiliations:** Lombardi Comprehensive Cancer Center, Georgetown University Medical Center, Washington, DC, United States of America; Duke University School of Medicine, UNITED STATES

## Abstract

Clofarabine, an FDA approved purine analog, is used in the treatment of relapsed or refractory acute lymphoblastic leukemia. Clofarabine acts by inhibiting DNA synthesis. We demonstrated that clofarabine may have a novel function though inhibiting CD99, a transmembrane protein highly expressed on Ewing Sarcoma (ES) cells. CD99 is a validated target in ES whose inhibition may lead to a high therapeutic index for patients. Here we present additional data to support the hypothesis that clofarabine acts on CD99 and regulates key signaling pathways in ES. Cellular thermal shift assay indicated a direct interaction between clofarabine and CD99 in ES cell lysates. Clofarabine induced ES cell death does not require clofarabine’s conversion to its active form by deoxycytidine kinase. A phosphokinase array screen with clofarabine and a CD99 blocking antibody identified alterations in signaling pathways. CD99 inhibition with clofarabine in ES cells caused rapid and sustained phosphorylation of ERK, MSK, and CREB. However, activation of this pathway did not correlate with clofarabine induced ES cell death. In summary, we demonstrated that clofarabine may activate ERK, MSK, and CREB phosphorylation through CD99 within minutes, however this paradoxical activation and subsequent ES cell death requires additional investigation.

## 1. Introduction

CD99 is an integral membrane protein, related to the variety of cellular mechanisms including cell differentiation, migration, adhesion, and apoptosis. These diverse mechanisms support a role for CD99 in immune system development, immune response in inflammation, and the pathogenesis of cancer [1]. A large group of tumors including malignant gliomas, leukemias, lymphomas, small round cell sarcomas, and mesenchymal chondrosarcomas have increased levels of CD99 expression [2–5]. Clear, strong membrane expression of CD99 is used as a diagnostic marker to distinguish ES from other small round cell tumors [6, 7].

Ewing sarcoma is a malignant small round cell tumor mainly affecting children and adolescents. Expression of CD99 and specific translocations between the *TET/FET* and *ETS* family genes accepted as diagnostic markers for ES [7, 8]. CD99 has a pivotal role in ES tumor pathogenesis by regulating tumor cell differentiation and proliferation [3, 9]. Knockdown of CD99 expression in ES cells suppressed proliferation in vitro and reduced growth of xenograft tumors in mice [10]. Blocking CD99 expression by shRNA caused neural differentiation and growth inhibition with block at G2/M phase in ES cells [11]. The vital role of CD99 in ES as well as its paucity of membranous expression in non-tumor cells support CD99 as a therapeutic target.

We recently reported that clofarabine and cladribine may have a new molecular mechanism by directly binding to CD99 on cell surface, which is independent of inhibiting DNA synthesis by its 5`-triphosphate form [12]. Clofarabine and cladribine are deamination resistant analogs of deoxyadenosine. Both drugs are FDA approved for treating refractory or relapsed acute myeloid leukemia (AML) and acute lymphoblastic leukemia (ALL) [13–15].

Intracellular signal pathways associated with ES cell death following CD99 binding by clofarabine remain to be clarified. Therefore, we investigated signaling pathways in ES cells following inhibition of CD99 with clofarabine. We found that clofarabine treatment resulted with ES cell death while activating phosphorylation of extracellular signal‑regulated protein kinase1/2 (ERK1/2), mitogen and stress activated kinases 1/2 (MSK1/2) and cAMP response element-binding protein (CREB) phosphorylation. Our work continues to identify new mechanisms of clofarabine through CD99 action that could lead to novel targeting of ES.

## 2. Materials and methods

### 2.1. Cell culture and CD99 blocking

Ewing sarcoma (ES) cell lines; STA-ET-7.2, TC-71, and RD-ES were cultured in RPMI (Gibco) supplemented with 10%FBS (Gibco). TC-32 and A4573 cells were cultured RPMI supplemented with 10%FBS and 10 mmol/L HEPES (Gibco). Osteosarcoma (OS) cell lines; MG-63, U2-OS and SAOS-2 cells were grown in DMEM (Gibco) with 10%FBS.

Antibody treatments were done using 15μg/ml CD99 mAb clone [HO36-1.1] (Abcam, #Ab212605), CD99 mAb clone (12E7) (Dako, #M3601), CD99 mAb clone (013) (Invitrogen, #MA5-12287), IgG (Invitrogen #MA1-10405), and IgM (Abcam, Ab18400). The X-tremeGENE siRNA Transfection Reagent (Sigma, Aldrich, #04 476 115 001) was used for siRNA experiments. Transfection carried out according to manufacturer’s protocol. Briefly, A4573 cells prepared at 30–50% confluence, X-tremeGENE siRNA transfection reagent with serum-free OptiMEM I medium (Gibco) and siRNA were mixed and incubated for 15–20 minutes at room temperature. After incubation, the reagent was added to the plates drop wise and swirled gently and incubated under standard culture conditions.

### 2.2. Cell viability assays

The effects of inhibitors on ES or OS cell viability was analyzed with WST-1 cell proliferation reagent (Roche, #11644807001). Briefly, cells were seeded on to 96-well plates and incubated for 24 hours. After 24 hours of incubation, the medium was replaced with the one containing inhibitors or DMSO. Cells were incubated for additional 24 to 48 hours. When the incubation period completed, WST-1 added to each well (10uL per 100uL media) and incubated at dark for 3 hours 37°C. Absorbance was measured at 440nm and 650nm with a Synergy H4 hybrid microplate reader (Biotek). All experimental groups had three replicates for each treatment and all experiments repeated three times.

### 2.3. Cellular thermal shift assay

Cellular Thermal Shift Assay (CETSA) was performed with TC-71 cells to monitor the drug-protein binding through quantification of the change in the thermal denaturation temperature. Whole cell lysates were diluted to 1.5μg/μl and split into two groups for Clofarabine (CLF) or DMSO treatment. Lysates were incubated for 30 min at room temperature. Proteins were denatured with Veriti 96-well Thermal Cycler for 3 min at different temperatures ranging from 46°C to 85°C and centrifuged at 16000 X *g* for 30 min at 4°C. Supernatants were collected and western blotting was performed with 30μg load per well.

### 2.4. Phospho-Kinase Array

The Human Phospho-Kinase Array (R&D Systems, #ARY003B) was used for analyzing phosphorylation of 43 kinases and 2 related proteins. STA-ET-7.2 cells treated with clofarabine, DMSO, CD99 antibody (Abcam, #ab212605) and IgM (Abcam, #ab18400) for 24 hours. Data analysis was done using image analysis software ImageJ v1.48 [16].

### 2.5. Western blotting

Western blotting experiments were done as previously described [17] using following primary antibodies; anti-ERK1/2 (#9107S), anti-pERK1/2 (#4370S), anti-MSK1 (#3489), anti-MSK2 (#3479), anti-CREB (#4820S), anti-pCREB (#9198S), anti-STAT-3 (#9139S), anti-pSTAT-3 (#9131S) (Cell Signaling Technology), anti-pMSK1/2 (RD Systems, #MAB1094), anti-DCK (#Ab96599), and anti-actin (Abcam, #ab20272); secondary antibodies anti-mouse or anti-rabbit (Amersham ECL, #NA931V, #NA934V). Chemiluminescences of blots were detected with Fujifilm LAS-3000 imaging system. The experiments were independently performed at least three times.

### 2.6. Statistical analysis

Statistical analyses were performed by GraphPad Prism (v8.4.3). Unpaired Student’s t-tests were used for analysis. Data was expressed as mean ± standard deviation of three replicates. A value of p<0.05 was considered significant.

## 3. Results

### 3.1. Clofarabine has a novel function on the plasma membrane that does not require activation by phosphorylation

**Fig 1:**
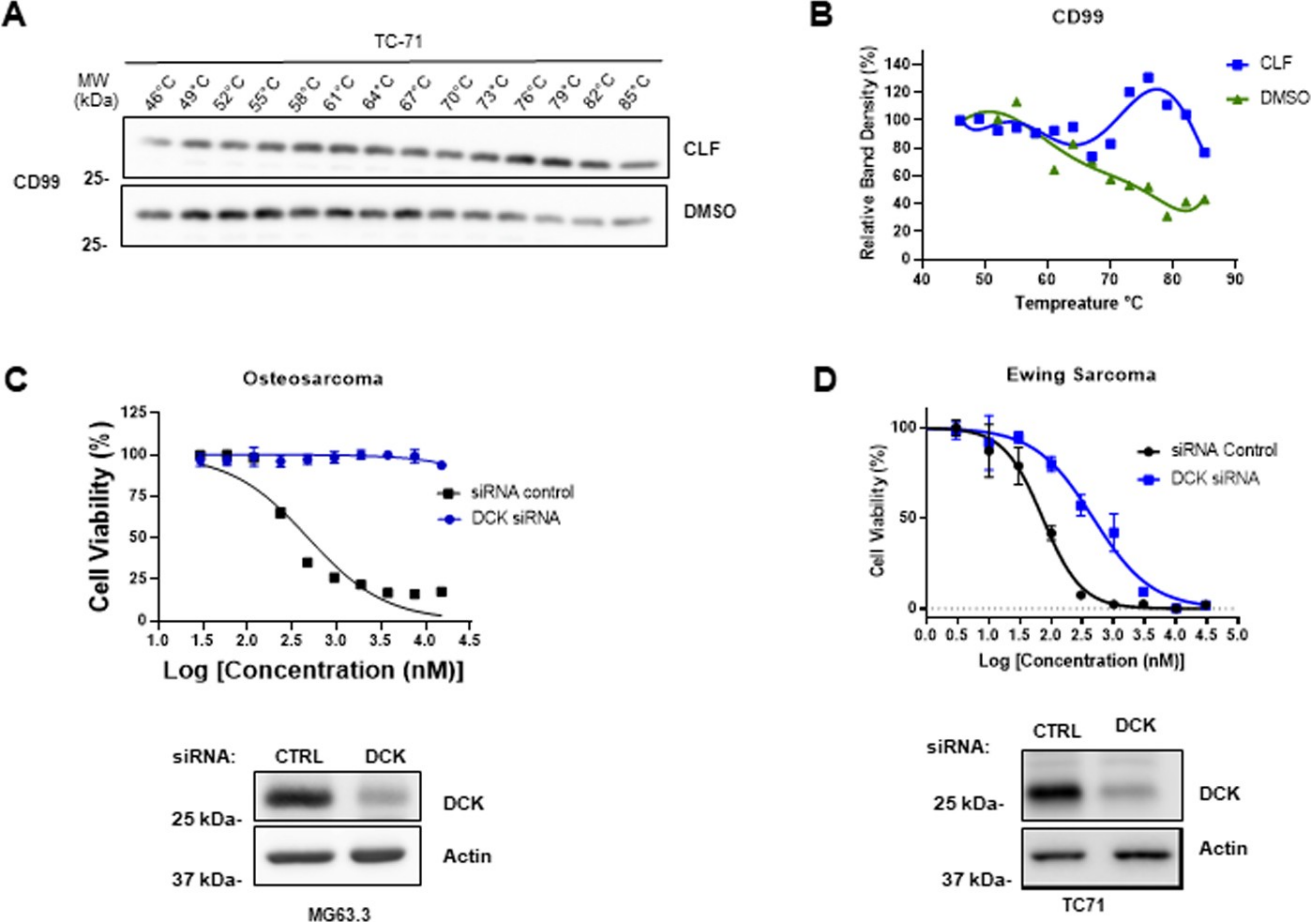
Clofarabine can bind CD99 and inhibit ES cell growth as a prodrug. **(A)** Thermal stability of CD99 protein was tested in the presence of clofarabine (CLF) by western blotting. CD99 stabilized by clofarabine stays in solution at higher temperatures; **(B)** Graph represents band density of CD99 from panel a. Densities of bands were measured with ImageJ and analysis was done using GraphPad Prism. **(C, D)** MG63.3 OS and TC-71 ES cell lines were transiently transfected with control (black lines) or DCK siRNA (blue lines). DCK protein expression was confirmed by western blot. Reduced DCK expression resulted in complete resistance to clofarabine in OS cells but not in ES cells.

We demonstrated that clofarabine can directly bind to purified recombinant CD99 (both from bacterial and mammalian sources) in a surface plasmon resonance assay (Bioacore) [12]. Here we further confirmed the same interaction by studying clofarabine binding to endogenous CD99 protein in ES cell (TC-71) lysate. Cellular thermal shift assay (CETSA) allows detection of drug-bound stabilized target protein in solution at elevated temperatures [18, 19]. In this assay free protein denatures and precipitates at a specific temperature unique to each protein. Direct binding of a small molecule generally results in enhanced stability and increased temperature for denaturation. Unlike the unbound protein, which denatured and precipitated from the soluble fraction, CD99 stabilized by clofarabine stayed in solution at higher temperatures (Fig 1A and 1B). Therefore, the increased thermostability of endogenous CD99 in ES cell lysates incubated with clofarabine support the data with recombinant CD99 protein.

We further provide evidence for clofarabine acting on plasma membrane through a functional assay. In order to inhibit DNA synthesis clofarabine and cladribine must be converted to their active forms, clofarabine-5`-triphosphate and cladribine-5`-triphosphate, respectively. Phosphorylated forms of these compounds cannot penetrate the plasma membrane [20–22]. Therefore, clofarabine and cladribine are administered as prodrugs, which are phosphorylated intracellularly by deoxycytidine kinase (DCK) to their active forms [23, 24]. In order to further support the hypothesis that clofarabine has a novel function through a cell surface protein CD99, we inhibited expression of DCK in both ES cells (TC-71 and TC-32) that express CD99 and OS cells (MG63.3 cell line) that express no detectable CD99. In the OS cell line inhibiting DCK protein expression resulted in complete resistance to clofarabine, which suggest that the observed cell death in OS cells was entirely due to inhibition of DNA synthesis by clofarabine-5`-triphosphate (Fig 1C). In contrast, lack of DCK protein only provided a shift to right of the IC_50_ curve in ES cell line, suggesting that the cell death observed in ES cells was a combined effect of both DNA synthesis inhibition by clofarabine-5`-triphosphate and CD99 inhibition by clofarabine (Fig 1D and S1A Fig). The difference in amplitude of IC_50_ shift to right following DCK siRNA treatment in two cell lines (TC71 and TC32) did not correlate with levels of CD99 expression in these cells (S1C Fig).

### 3.2. Inhibition of CD99 by clofarabine activates protein kinase pathways

**Fig 2:**
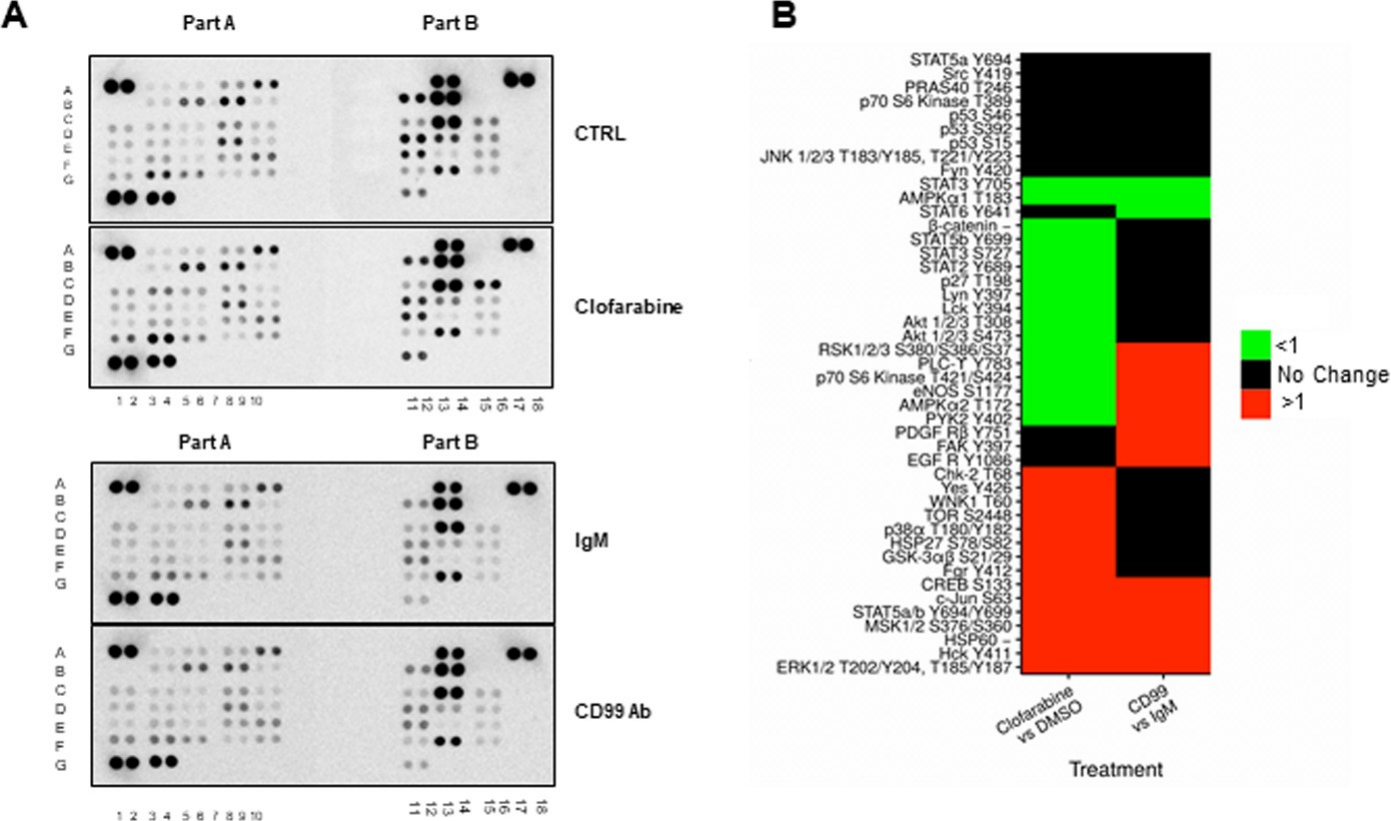
Phosphokinase array analysis of STA-ET.7.2 cells after treatment with clofarabine and CD99 antibody. **(A)** Phosphokinase screening of cells treated with clofarabine (0.6 μM) and DMSO (upper pair) or CD99 antibody (15 μg/ml), and IgM (15 μg/ml) (lower pair). Protein coordinates of the kinase array are given in S1 Fig; **(B)** Analysis of band intensity after treatment with clofarabine and CD99 antibody is given. Red color indicates increased phosphorylation, black color indicates no change and green color indicates decreased phosphorylation compared to control in each group. ERK1/2, MSK1/2, CREB, c-Jun, HSP60, Hck and STAT5 phosphorylation levels were increased while STAT3 and AMPKα1 were decreased for both treatment groups.

The vast majority of transmembrane proteins lead to regulation of cytoplasmic signaling cascades. In order to evaluate which signaling pathways are regulated following inhibition of CD99 in ES cell lines, we probed a phosphokinase array. The array is a membrane-based immunodetection method that allowed us to screen site-specific phosphorylation of 43 kinases and 2 related proteins. An ES cell line expressing high levels of CD99, STA-ET-7.2, was treated with clofarabine and an anti-CD99 antibody to inhibit CD99 function in regular growth conditions. Following 30 min of treatment, cell lysates were collected and probed using the phosphokinase array membrane. DMSO treated cells were used as the control for clofarabine and total mouse IgM treated cells were used as control for anti-CD99 antibody groups (Fig 2 and S2 Fig). The phosphokinase array contains two spots for each target protein and replicate membranes are used for control and experimental groups. A positive result required a signal in both protein spots. Clofarabine treatment showed increased phosphorylation of 15 proteins, and decreased phosphorylation of 17 proteins (Fig 2A, upper panel). CD99 targeting antibody (clone [HO36-1.1]) demonstrated increased phosphorylation of 16 proteins and decreased phosphorylation of 3 proteins when compared to the negative control total IgM antibody. We focused on the phosphorylation events that were upregulated or down regulated by both clofarabine and anti-CD99 treatments (Fig 2B). We concluded that if a specific phosphorylation event is induced by both clofarabine and anti-CD99 treatments, it has a higher probability of being directly regulated by CD99 protein on the plasma membrane. Phosphorylation of 7 proteins (ERK1/2, MSK1/2, CREB, c-Jun, HSP60, Hck, and STAT-5) were increased in response to both clofarabine and anti-CD99 antibody in STA-ET-7.2 ES cell line. -Based on these observations we show that CD99 inhibition, by two different approaches in ES cells, leads to the common pattern of increased phosphorylation of ERK1/2, MSK1/2, CREB, c-Jun, HSP60, Hck, and STAT-5 and reduced phosphorylation of STAT-3 and AMPKα1.

### 3.3. CD99 inhibition by clofarabine induce phosphorylation of ERK1/2, MSK1/2, and CREB

**Fig 3:**
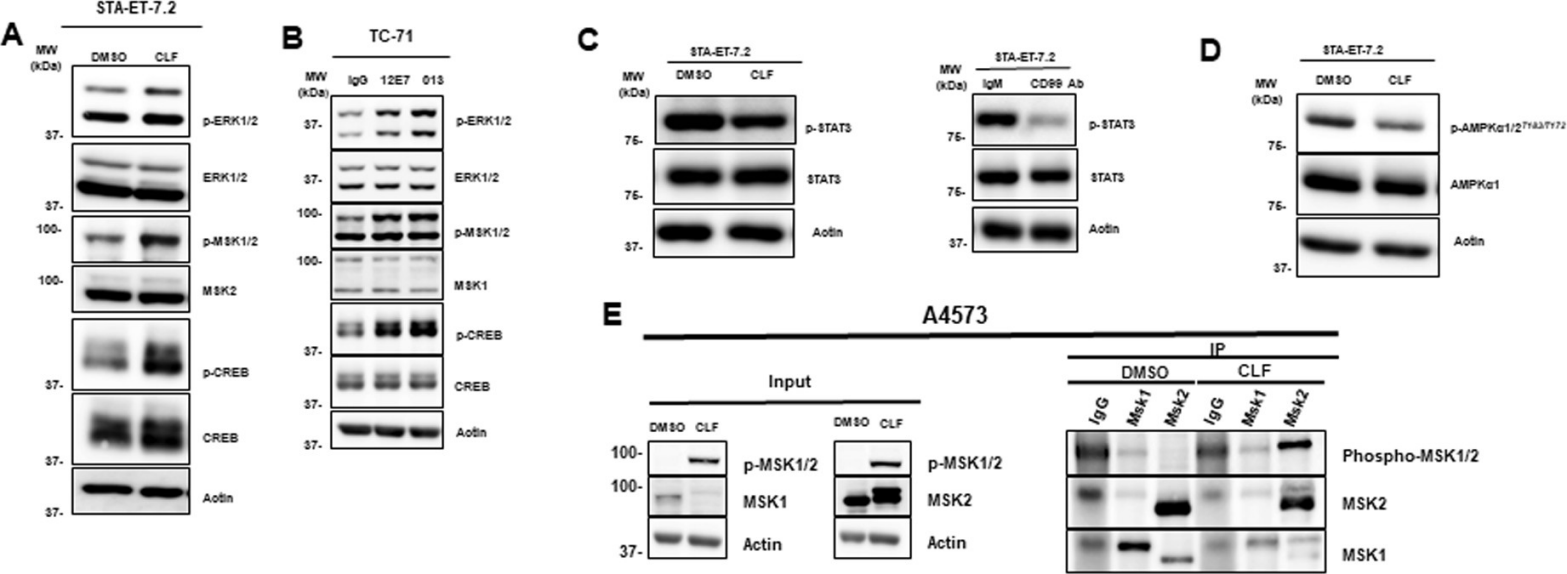
Clofarabine and CD99 antibodies cause specific protein phosphorylation in ES cell lines. **(A)** STA-ET-7.2 cells treated with clofarabine and western blot analyses were done by phosphospecific antibodies. Phosphorylation of ERK1/2, MSK1/2 and CREB levels were increased with clofarabine treatment; **(B)** TC-71 cells treated with two different C99 antibodies (15μg/ml) and phosphorylation changes were analyzed by immunoblotting. Similar to clofarabine treatment, phosphorylation of ERK1/2, MSK1/2 and CREB were increased; **(C, D)** STA-ET-7.2 cells treated with clofarabine, CD 99 antibody and decreased phosphorylation changes for STAT3 and AMPKα1 were validated by western blotting; **(E)** A4573 cells treated with clofarabine show increased level of pMSK1/2 and main phosphorylated protein is pMSK2.

In order to validate the phosphokinase array screening findings, we performed immunoblot analysis with fresh cell lysate and kinase specific antibodies in ES cells. Of the 7 common proteins (ERK1/2, MSK1/2, CREB, c-Jun, HSP60, Hck, and STAT-5) with increased phosphorylation in response to both clofarabine and anti-CD99 antibody in the phosphokinase array screening, we consistently observed increased phosphorylation of ERK1/2, MSK1/2, and CREB after clofarabine treatment (Fig 3A). We found that these effects were not specific to a single antibody product as levels of pERK1/2, pMSK1/2, and pCREB were also observed following treatment with two additional CD99 antibodies (Fig 3B). We also observed increase in c-Jun phosphorylation in response to clofarabine in multiple ES cell lines at both early and late time points. However, the increase in phosphorylation was correlating with increase in total c-Jun protein levels (S3A Fig). Therefore, we did not further investigate c-Jun in this study. None of the antibodies we tried gave reliable results for pHSP60, pHck, and pSTAT-5. Immunoblots for these three proteins were poor quality and not reproducible. Therefore, we were not able to validate the increased phosphorylation of these proteins in response to clofarabine or anti-CD99 antibodies. Reduced phosphorylation levels of AMPKα1 and STAT3 were also validated by western blotting (Fig 3C and 3D; S4A Fig).

Total and phospho-specific MSK antibodies we used in Fig 3A detect both MSK1 and MSK2 proteins. Therefore, increased phosphorylation of either MSK1 or MSK2 in the total cell lysate could be responsible for the positive signal in Fig 3A. In order to identify which isoform of MSK is phosphorylated in response to clofarabine treatment, we first immunoprecipitated MSK1 and MSK2 with isoform specific antibodies and then performed an immunoblotting with A4573 and TC-32 cell lines (Fig 3E and S4B Fig). The results confirmed that the main isoform that was phosphorylated in ES cells in response to clofarabine was MSK2.

### 3.4. Clofarabine induced phosphorylation is rapid and sustained

**Fig 4:**
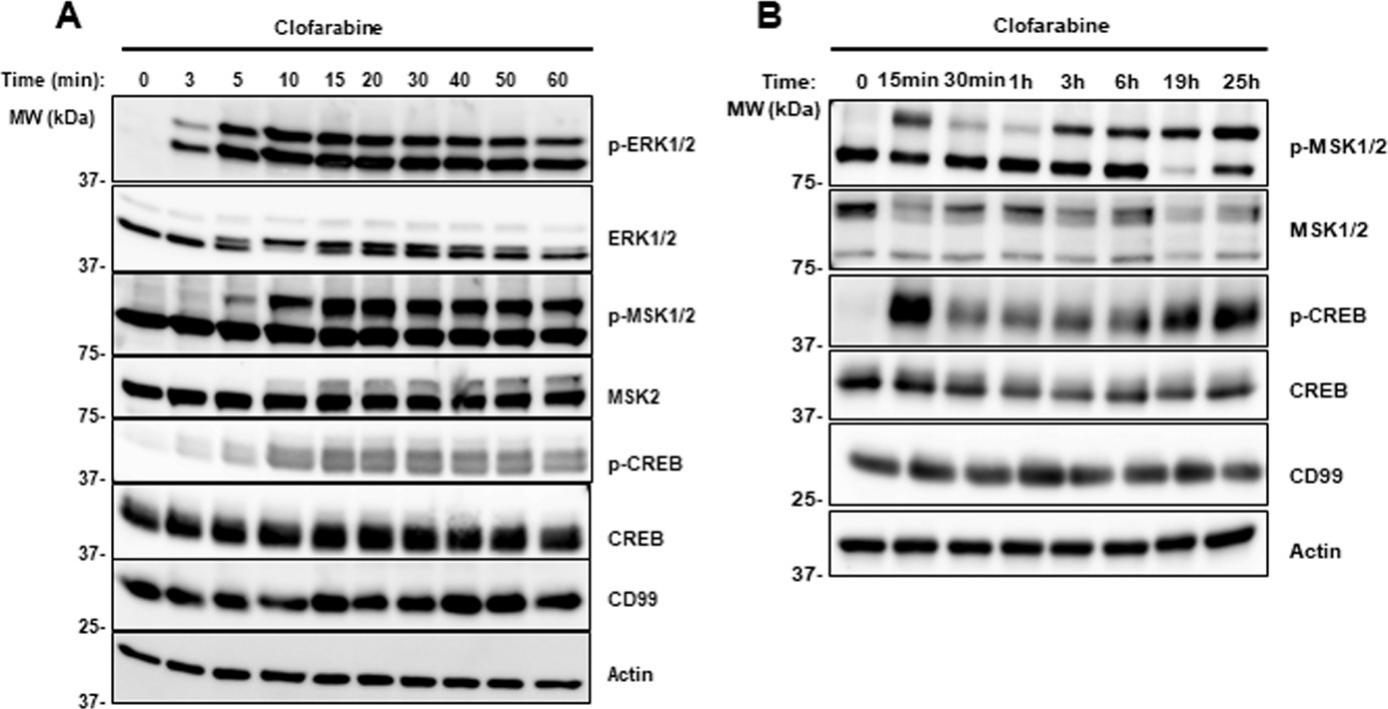
Time course of phosphorylation changes after clofarabine treatment of STA-ET-7.2 cells. **(A)** STA-ET-7.2 cells treated with 0.5μM clofarabine and total cell lysates collected at different time points. Western blot results showed increased phosphorylation starting as early as 3 minutes; **(B)** phosphorylation changes were sustained through 25 hours with a reduction around one hour. Actin was used as loading control.

We hypothesize that clofarabine induced cell death in ES cells is a combined effect of inhibiting CD99 on plasma membrane and inhibiting DNA synthesis following activation by DCK that was supported by the findings in Fig 1C and 1D. Since clofarabine treatment also induced phosphorylation of ERK1/2, MSK1/2, and CREB, it is possible that the alterations in phosphorylation of these proteins may be result of its ability to inhibit DNA synthesis or inhibit CD99. Since most membrane initiated signaling pathways are rapid responders, we evaluated phosphorylation status over time in order to further understand the clofarabine’s mechanism of action. ES cells STA-ET-7.2 were treated from 3 to 60 minutes followed by total protein extraction and immunoblot analysis for ERK1/2, MSK1/2, and CREB. We observed a rapid induction of phosphorylation events as early as 3 min following clofarabine treatment (Fig 4A). The phosphorylation effect appeared to be biphasic. We then repeated the experiment with longer time points stretching out to 25 hours. Following the initial induction, there was a reduction around 1h and sustained increase through 25h (Fig 4B). The rapid induction that was observed in STA-ET-7.2 cells was also replicated in A4573 cell line (S3B Fig). These findings demonstrated that clofarabine caused a swift and sustained phosphorylation changes of signal pathway components following clofarabine treatment, which further support the novel role of clofarabine on the plasma membrane.

### 3.5. Clofarabine but not cytarabine induced phosphorylation of ERK1/2, MSK1/2, and CREB

**Fig 5:**
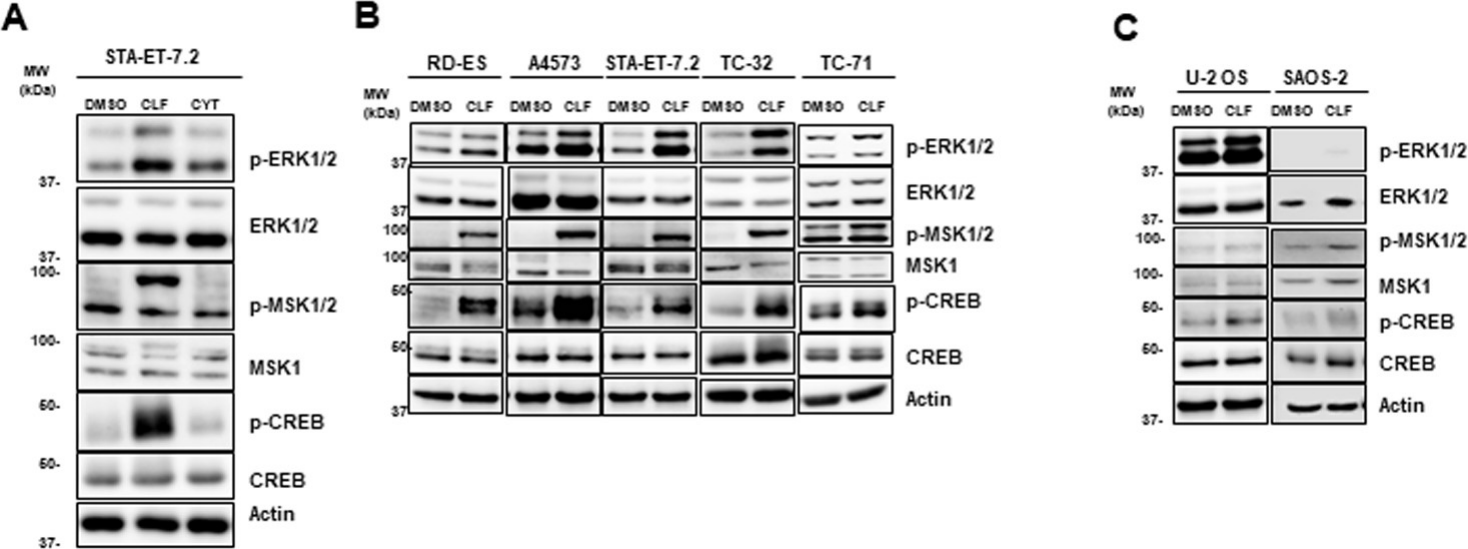
Phosphorylation changes after treatment for different ES and OS cell lines. **(A)** Cytarabine’s effect on STA-ET.7.2 cells compared to the clofarabine treatment. Cells were treated with clofarabine, cytarabine or DMSO. Immunoblot analysis showed cytarabine did not alter phosphorylation of ERK1/2, MSK1/2 and CREB; **(B)** Immunoblot results of phosphorylation changes after clofarabine treatment of different ES cell lines; clofarabine caused increased phosphorylation of ERK1/2, MSK1/2 and CREB in all five ES cell lines; **(C)** Clofarabine treated OS cell lines did not show significant increase in phosphorylation of ERK1/2, MSK1/2 and CREB. Actin was used as the loading control for all experiments. (CLF: Clofarabine, CYT: Cytarabine).

We investigated a close analog of clofarabine, cytarabine. Cytarabine was proposed as a treatment for patients with ES, based on cDNA expression profile similarity [25], however, cytarabine clinical trials did not show benefit to the patients [26, 27]. Clofarabine and cladribine are purine analogs whereas cytarabine is a pyrimidine analog. In order to more fully evaluate the role of cytarabine on ES cells, we compared the effects of clofarabine and cytarabine on STA-ET-7.2 cells. Cytarabine did not activate ERK1/2, MSK1/2 or CREB phosphorylation (Fig 5A). In support of this finding, two additional cell lines, TC-71 and TC32 found no post-translational phosphorylation in this panel of proteins following cytarabine treatment (S3C Fig). Since DNA synthesis inhibition is a common pathway between purine and pyrimidine analogs the differences we observed between clofarabine and cladribine in protein phosphorylation are most likely due to specific inhibition of CD99 by clofarabine.

### 3.6. Clofarabine induces phosphorylation of ERK1/2, MSK1/2, and CREB through CD99 blockage in multiple ES cell lines but not in osteosarcoma cell lines

Previously, we showed that clofarabine suppress cell viability and induce apoptosis in ES cells but not in osteosarcoma (OS) cell lines [12]. OS cells were selected as negative control because they do not express CD99 and they are unresponsive to anti-CD99 antibodies. We hypothesized that the clofarabine treatment of OS cells will not change the phosphorylation pattern of selected signal pathway components. Therefore, we treated two OS cell lines (U2-OS and SAOS-2) with clofarabine for 24 hours and collected the total cell lysates. Western blot analysis showed that TC-71, TC-32, RD-ES, STA-ET-7.2 and A4573 cells showed significant increase in pERK1/2, pMSK1/2, and pCREB levels while U2-OS and SAOS-2 cells did not (Fig 5B and 5C). These findings supported our data that clofarabine caused signal pathway changes particularly on ES cell lines due to CD99 expression on their surface. Some of these experiments were repeated many times for multiple cell lines throughout the study and provided very reproducible results (15 times for STA-ET-7.2, 16 times for A4573, 3 times for TC-71, 5 times for TC-32). We hypothesized that the difference in biological response to clofarabine between ES cells and OS cells was due to presence of CD99 in ES cells and lack of CD99 in OS cells rather than a direct effect on DNA synthesis. To further support our hypothesis, we also silenced CD99 for A4573 cells with siCD99 and CRISPR/Cas system. After selecting CD99 silenced clones we treated cells with clofarabine and collected cell lysates. Western blot results showed that silenced cells did not showed increase in pERK1/2, pMSK1/2, and pCREB levels (S5 Fig).

### 3.7. ERK1/2, MSK1/2, and CREB phosphorylation does not appear to be required for clofarabine induced cell death in ES cells

Inhibition of CD99 by clofarabine or antibodies results in apoptosis of ES cells. Since we observed rapid activation of ERK1/2, MSK1/2, and CREB phosphorylation following CD99 inhibition, we hypothesized that activation of these pathways may be leading to cell death. In order to test this hypothesis, we used pharmacological inhibitors of MSK (SB747651A) and MEK (PD184352) to rescue clofarabine mediated ES cell death [28–31]. When SB747651A and PD184352 were tested on three different ES cell lines (TC-71, STA-ET-7.2 and A4573), they did not alter the IC_50_ values of clofarabine (S6 Fig). Therefore, we concluded that even though ERK1/2, MSK1/2, and CREB phosphorylation happens rapidly following CD99 inhibition, activation of this pathway is not responsible for ES cell death. Therefore, CD99 inhibition mediated cell death and activation of ERK1/2, MSK1/2, and CREB phosphorylation appears to be true, true, unrelated observations. It is however still possible that activation of ERK1/2, MSK1/2, and CREB phosphorylation may be important for regulation of ES cell differentiation by CD99.

## 4. Discussion

The surface expression of CD99 on ES tumor cells is both a diagnostic marker and potential therapeutic target. CD99 antibodies, RNA interference, and small molecules were used for targeting CD99 [9, 32–34]. We reported the screening of a small molecule library to identify molecules that can directly bind to CD99 and result in ES cell death *in vitro* and *in vivo* [12]. Clofarabine is an FDA approved nucleoside analog and its mechanism of action is to inhibit DNA synthesis and cause apoptosis in leukemia cells [35]. In this study we aimed to describe changes in intracellular signal pathways in ES cells following CD99 inhibition with clofarabine. We found that pERK1/2, pMSK1/2, and pCREB levels are elevated with both anti-CD99 antibody and clofarabine treatment. Parallel to our findings Rocchi et. al. showed that blocking CD99 with shRNA systems caused increased ERK1/2 phosphorylation and neural differentiation of ES cells [11].

We evaluated a protein kinase array to analyze a broad spectrum of kinases in ES cells following CD99 inhibition by a small molecule or an antibody. We observed increased activation of ERK1/2, MSK1/2, CREB, c-Jun, HSP60, HCK, and STAT5 phosphorylation, which are related to the cell survival, apoptosis and cell stress response [36]. ERK1/2 activation can lead to resistance to chemotherapy and inhibition of apoptosis depending on cell type [37]. Increased pERK1/2 levels seen in ES cells with clofarabine treatment may be a cellular response to trying to overcome the cytotoxic effect. ERK1/2 activation leads to phosphorylation of MSK1/2, an activator of CREB, which can cause the rapid response to a variety of cellular responses related to cell growth and differentiation [29, 38, 39]. Increased phosphorylation of pERK1/2, pMSK1/2, and pCREB was seen in different ES cell lines but not in OS cell lines. While this manuscript was in submission process, a recent work established a potential ligand for CD99. Zhou et al, demonstrated that GDF6 (BMP13) prodomain can bind to CD99 and inhibit Src activity through C-terminal Src kinase (CSK) [40]. It would be very informational to evaluate if clofarabine can inhibit GDF6 binding to GDF6 and reverse its outcome on SRC signaling. Our earlier work suggested that clofarabine also inhibits Src activity in ES cell lines and xenografts [12]. Clorafabine effect on Src was delayed compared to its effect on ERK activation. Therefore, it may be though an alternative mechanism.

To find a clear explanation of decreased ES cell viability and increased phosphorylation of ERK1/2, MSK1/2, and CREB after treatment with clofarabine we also used kinase inhibitors targeting pERK1/2, pMSK1/2, and pCREB axis but cell viability did not change. MSK has a major role in regulating the signaling activities of the ERK pathway. In this pathway phosphorylated MSK activates transcription factor, CREB, which has pleiotropic activities in cell survival and differentiation [41–43]. Overexpression of these pathway components would let one to expect increased tumor growth. However, opposite to this expectation, we were observing decreased cell viability for different ES cell lines and reduced tumor growth in a xenograft model following clofarabine treatment.

The CD99 glycoprotein has two different variants CD99 type I and type II and cells could have these two variants or only one of them with a different ratio according to cell types. This phenomenon cause cell line based response and various signal pathway changes for CD99 inhibition of cells [44, 45]. Hahn et al. showed that three different MAPKs activated in response to CD99 ligation and ERK1/2 is the prominent one of these MAPKs [45]. Also, Vaikani et. al. showed that knocking down CD99 resulted with decreased phosphorylation of ERK for TH1 cells while no change for MOLM 13 cells [46]. Parallel to our report, Guerzoni et. al. showed that CD99 inhibition of ES patient derived cell lines addressed increased ERK phosphorylation [34]. However, none of these studies or our data presented in this manuscript could clearly explain CD99’s role in maintaining ES viability and its relationship to observed phosphorylation events.

In conclusion, we analyzed signal pathway changes for ES cells after blocking CD99 with clofarabine. Blocking CD99 with clofarabine decreased the ES cell viability and caused phosphorylation of ERK1/2, MSK1/2, and CREB. However, these phosphorylation changes did not seem to be critical for cell survival.

## Supporting information

S1 Fig DCK silenced cell viability. ES cells TC-32 **(A)** were transiently transfected with control (black lines) or DCK siRNA (blue lines). DCK protein expression was confirmed by western blot. Reduced DCK expression resulted in a shift to right of the IC_50_ curve. Total cell lysates form **(B)** Five ES cell lines were analyzed by western blot for CD99 expression. Actin was used as the loading control.
(TIF)

S2 Fig Coordinates of phosphokinase array used in Fig 2. (TIF)

S3 Fig **(A)** Total c-Jun and phosphorylates c-Jun levels were analyzed in clofarabine treated TC-71 and TC-32 cell lines. Increased phosphorylation levels were correlating with increase in total c-Jun protein levels. **(B)** Time course of phosphorylation changes after clofarabine treatment of A4573 cells showed rapid induction of MSK and CREB activation. **(C)** Cytarabine and Cladribine effects on TC-71 and TC-32 cells compared to the clofarabine treatment, cytarabine did not activate ERK1/2, MSK1/2 or CREB phosphorylation. Actin was used as the loading control for all experiments. (CLF: Clofarabine, CLD: Cladribine, CYT: Cytarabine).
(TIF)

S4 Fig Phosphorylation changes after clofarabine treatment. **(A)** After clofarabine treatment TC-71 and A4573 cell lines showed decreased STAT-3 phosphorylation. **(B)** TC-32 cells treated with clofarabine show increased level of pMSK1/2 and the main phosphorylated protein is pMSK2.
(TIF)

S5 Fig Phosphorylation changes after CD99 silencing. A4573 cells treated with clofarabine after silencing CD99 with siCD99 **(A)** and CRISPR/Cas system **(B)**; CD99 silenced cells did not show increased phosphorylation of ERK1/2, MSK1/2 and CREB.
(TIF)

S6 Fig MSK and MEK inhibitors did not alter IC_50_ values of ES cells. TC-71, STA-ET-7.2, and A4573 cell lines were treated with MSK and MEK inhibitors in the presence of clofarabine. IC_50_ values for MSK and MEK inhibitors did not change with clofarabine treatment. Analyses were done using GraphPad Prism. Data were expressed as mean ± standard deviation of three replicates.
(TIF)

S1 Raw images Raw data files of all western blots from figure data. (PDF)
