# Supplementary figures and images for: Clofarabine induces ERK/MSK/CREB activation through inhibiting CD99 on Ewing sarcoma cells
Source: PLoS One. 2021 Jun 16;16(6):e0253170. doi: 10.1371/journal.pone.0253170 (PMC8208565; doi:10.1371/journal.pone.0253170)

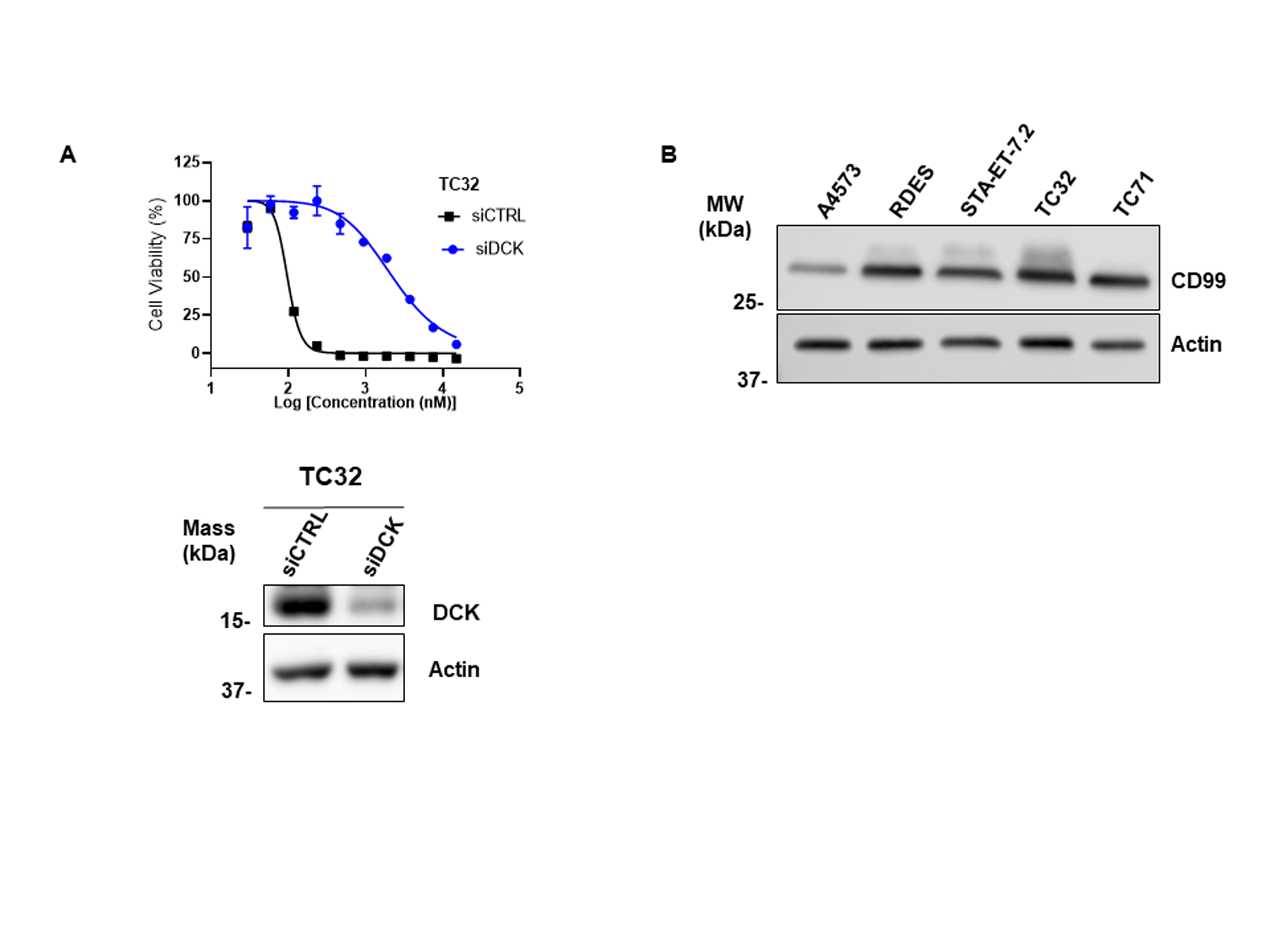

Supplement: S1 Fig — ES cells TC-32 (A) were transiently transfected with control (black lines) or DCK siRNA (blue lines). DCK protein expression was confirmed by western blot. Reduced DCK expression resulted in a shift to right of the IC50 curve. Total cell lysates form (B) Five ES cell lines were analyzed by western blot for CD99 expression. Actin was used as the loading control. (TIF) [file pone.0253170.s001.tif]

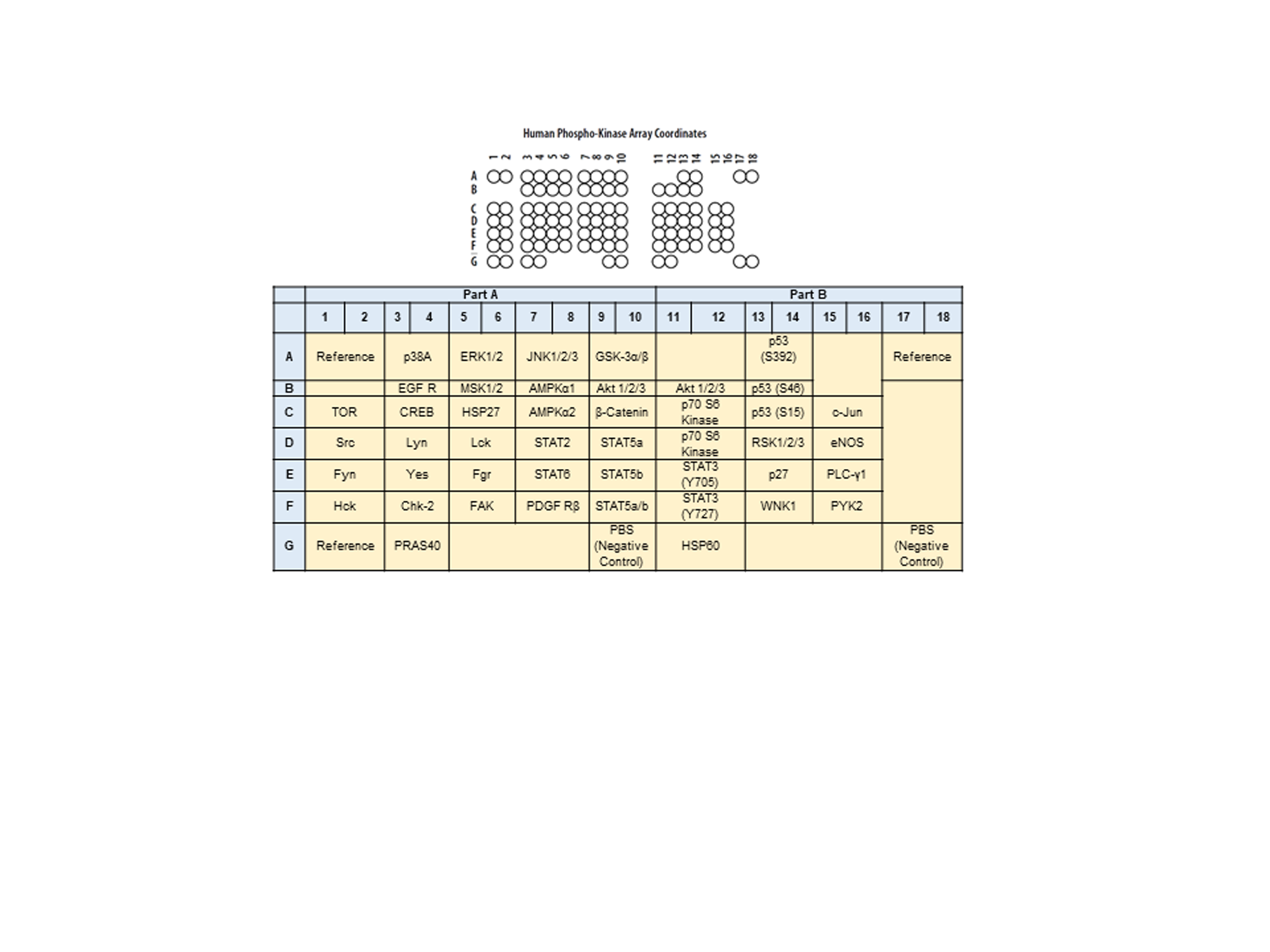

Supplement: S2 Fig — (TIF) [file pone.0253170.s002.tif]

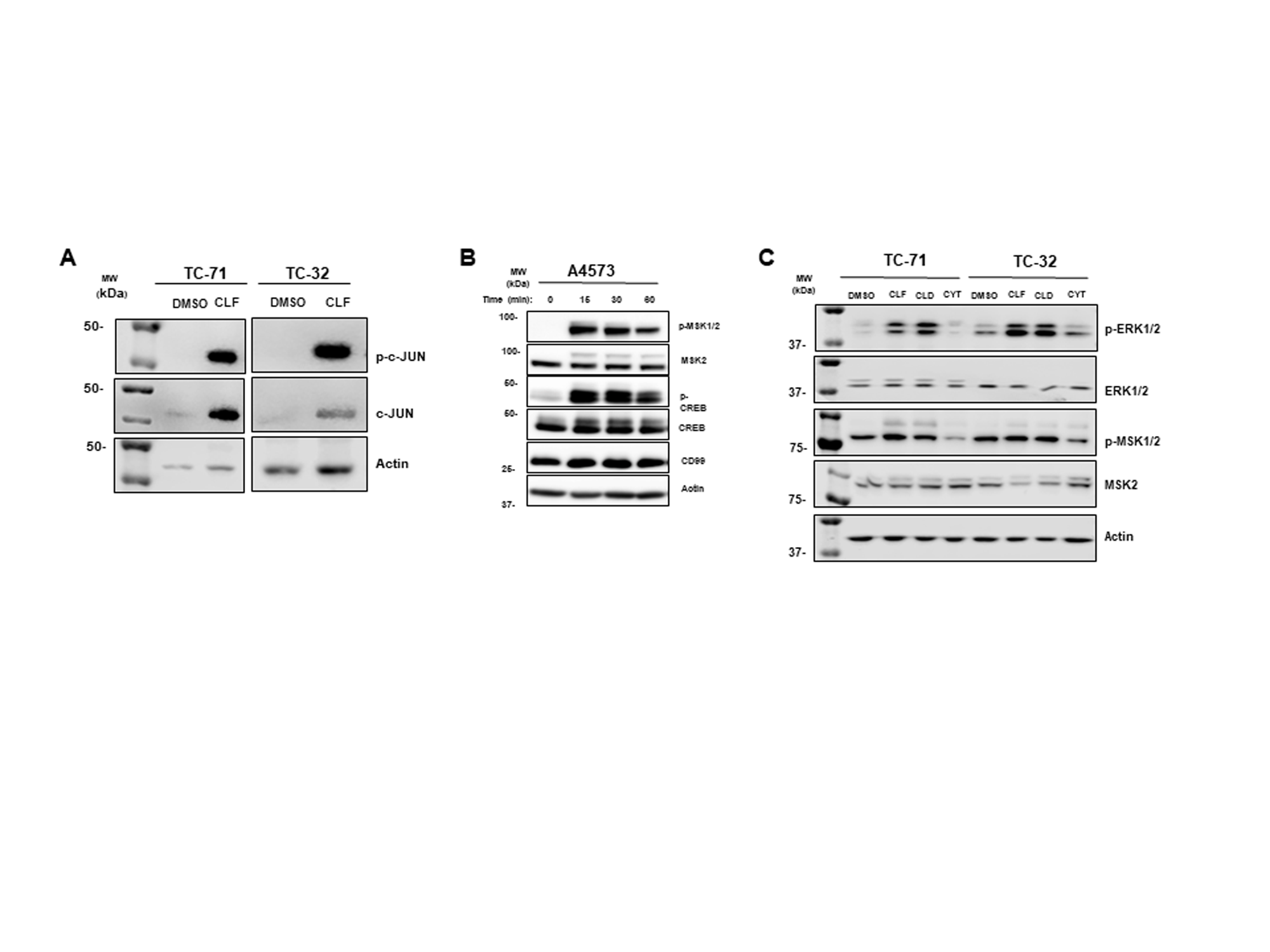

Supplement: S3 Fig — (A) Total c-Jun and phosphorylates c-Jun levels were analyzed in clofarabine treated TC-71 and TC-32 cell lines. Increased phosphorylation levels were correlating with increase in total c-Jun protein levels. (B) Time course of phosphorylation changes after clofarabine treatment of A4573 cells showed rapid induction of MSK and CREB activation. (C) Cytarabine and Cladribine effects on TC-71 and TC-32 cells compared to the clofarabine treatment, cytarabine did not activate ERK1/2, MSK1/2 or CREB phosphorylation. Actin was used as the loading control for all experiments. (CLF: Clofarabine, CLD: Cladribine, CYT: Cytarabine). (TIF) [file pone.0253170.s003.tif]

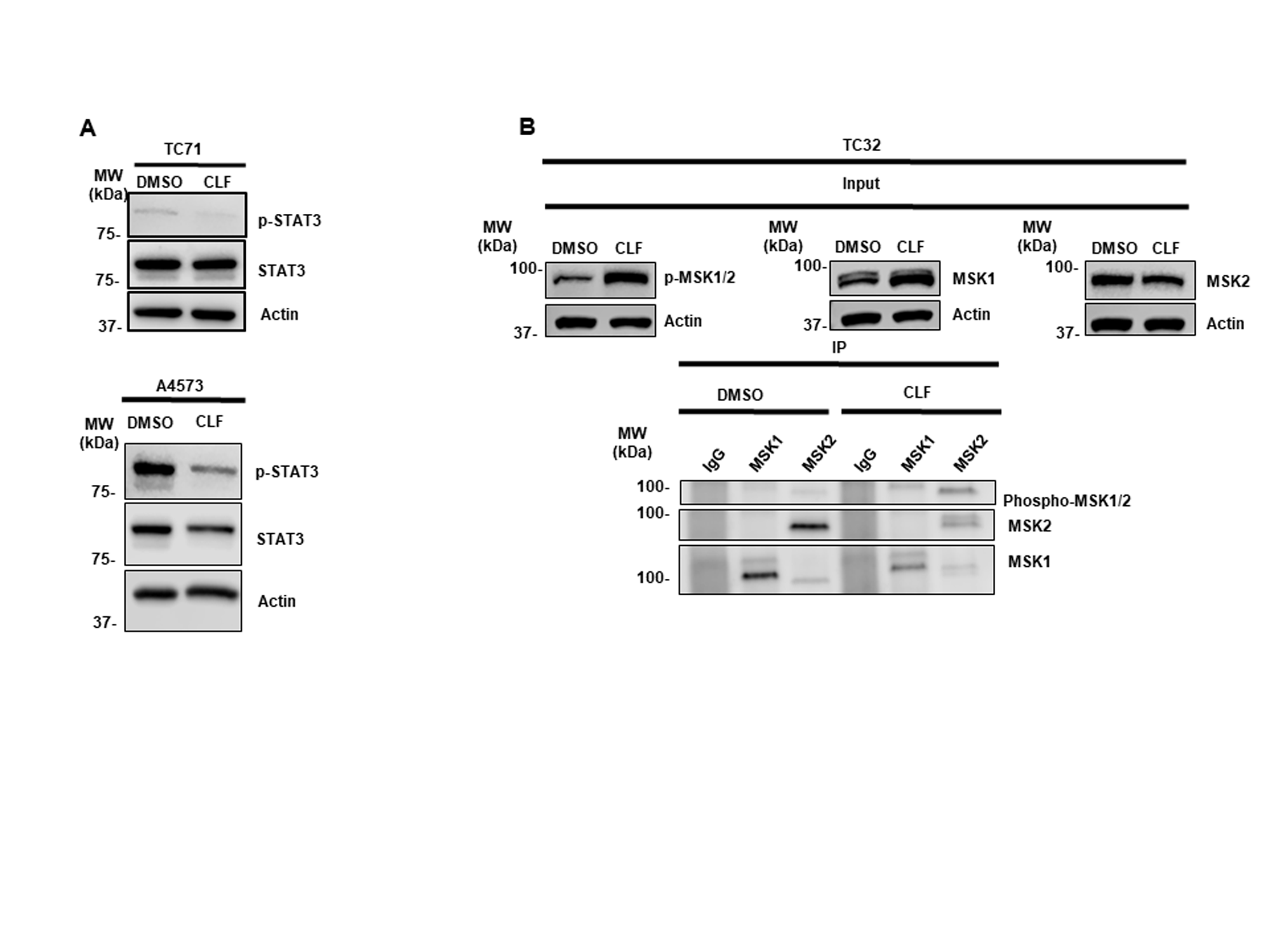

Supplement: S4 Fig — (A) After clofarabine treatment TC-71 and A4573 cell lines showed decreased STAT-3 phosphorylation. (B) TC-32 cells treated with clofarabine show increased level of pMSK1/2 and the main phosphorylated protein is pMSK2. (TIF) [file pone.0253170.s004.tif]

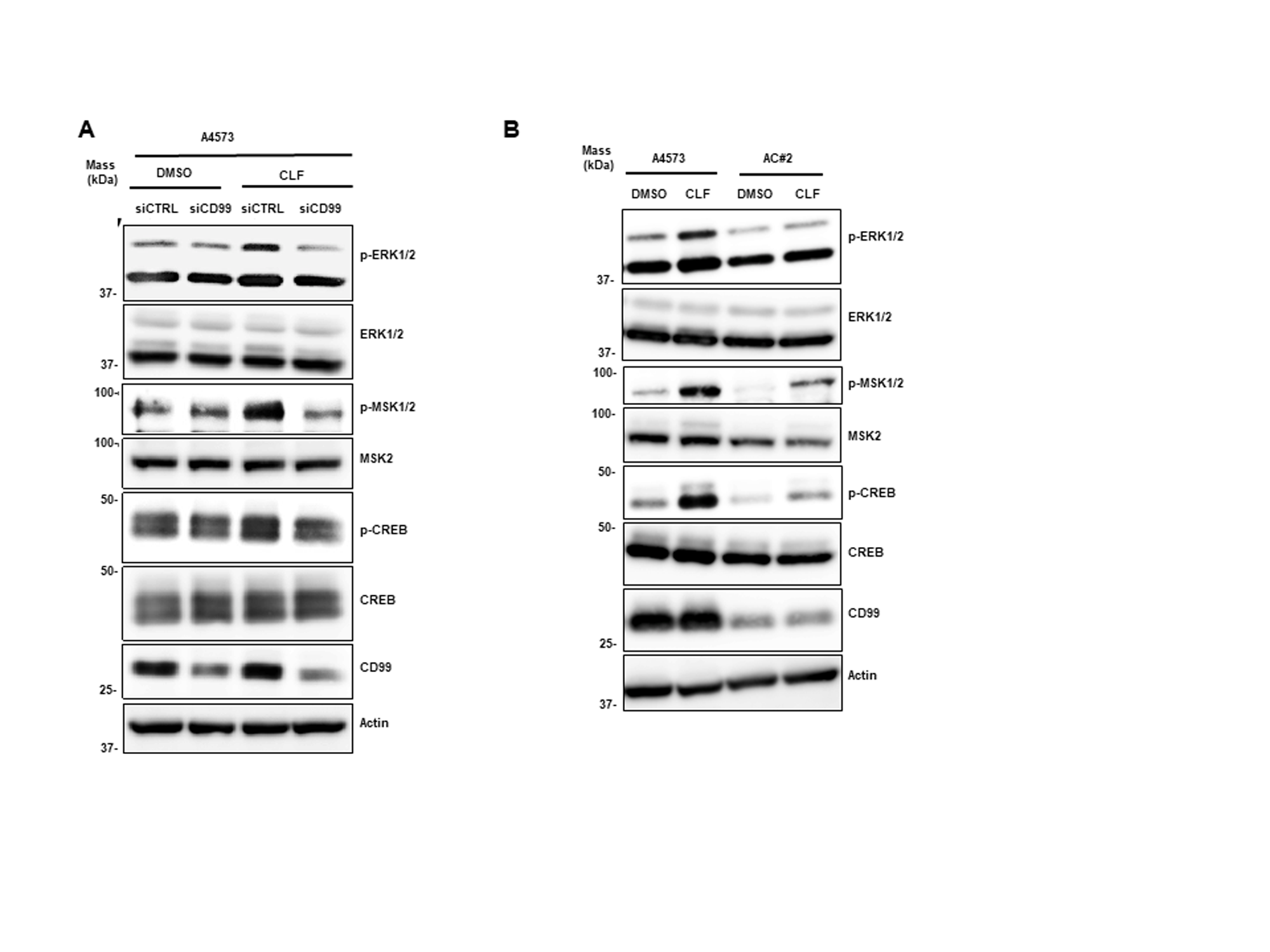

Supplement: S5 Fig — A4573 cells treated with clofarabine after silencing CD99 with siCD99 (A) and CRISPR/Cas system (B); CD99 silenced cells did not show increased phosphorylation of ERK1/2, MSK1/2 and CREB. (TIF) [file pone.0253170.s005.tif]

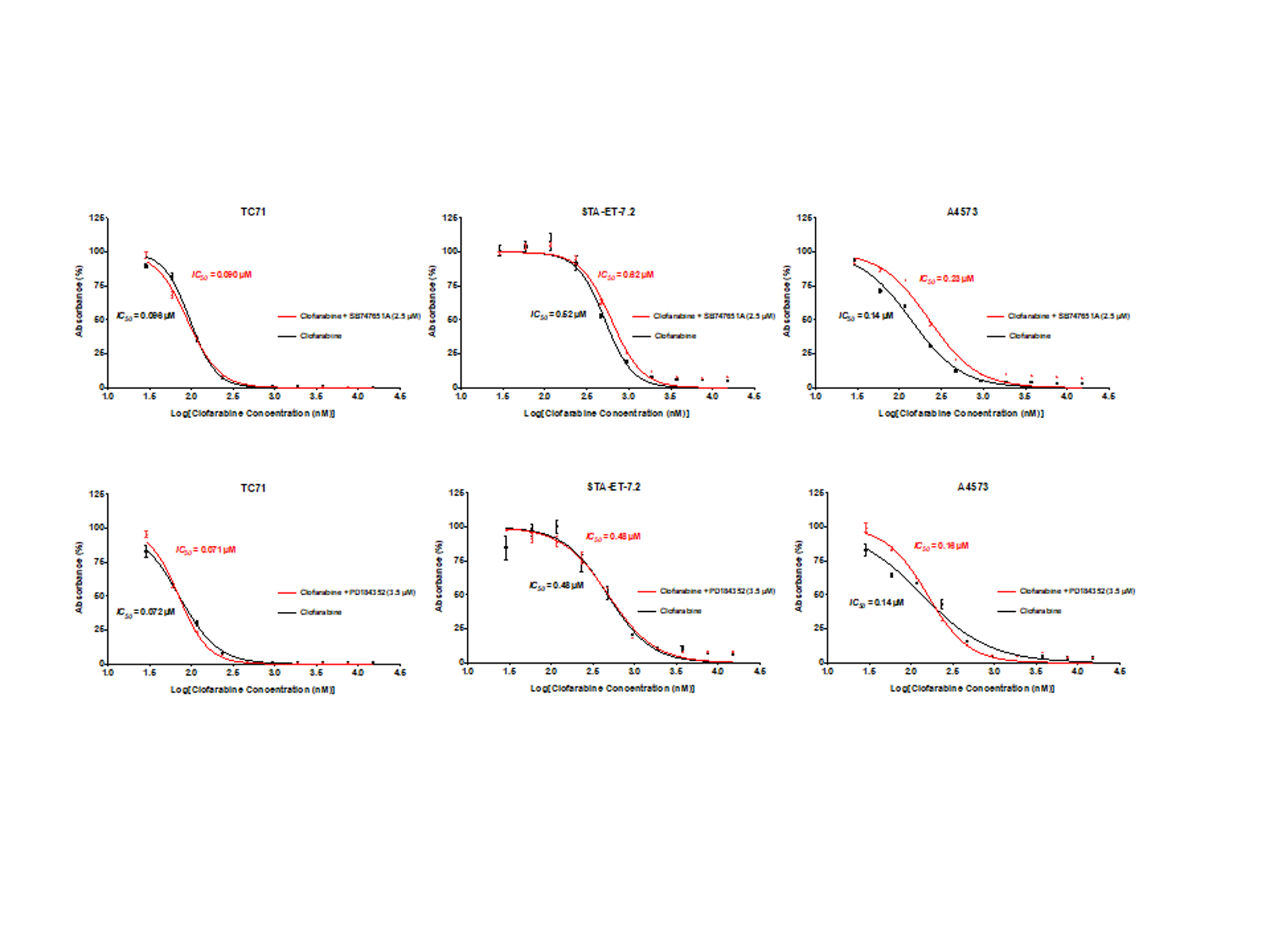

Supplement: S6 Fig — TC-71, STA-ET-7.2, and A4573 cell lines were treated with MSK and MEK inhibitors in the presence of clofarabine. IC50 values for MSK and MEK inhibitors did not change with clofarabine treatment. Analyses were done using GraphPad Prism. Data were expressed as mean ± standard deviation of three replicates. (TIF) [file pone.0253170.s006.tif]
