# Supplementary material for: Clofarabine induces ERK/MSK/CREB activation through inhibiting CD99 on Ewing sarcoma cells
Source: PLoS One. 2021 Jun 16;16(6):e0253170. doi: 10.1371/journal.pone.0253170 (PMC8208565; doi:10.1371/journal.pone.0253170)
Supplement: S1 Raw images — (PDF) [file pone.0253170.s007.pdf]

Figure 1A

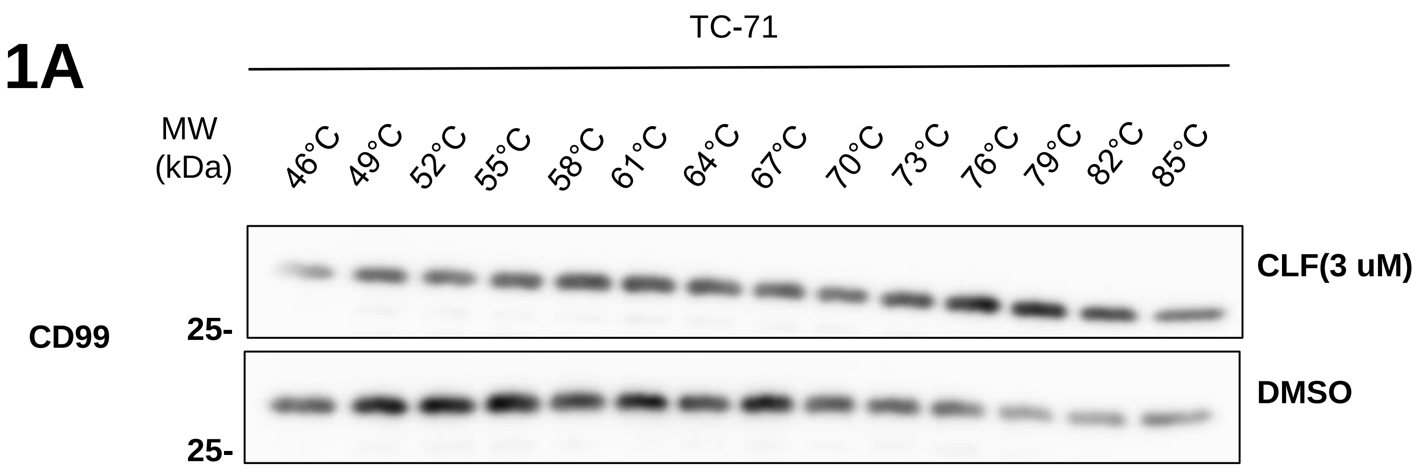

TC-71
MW (kDa)
46°C 49°C 52°C 55°C 58°C 61°C 64°C 67°C 70°C 73°C 76°C 79°C 82°C 85°C
CD99
25-
CLF(3 uM)
25-
DMSO

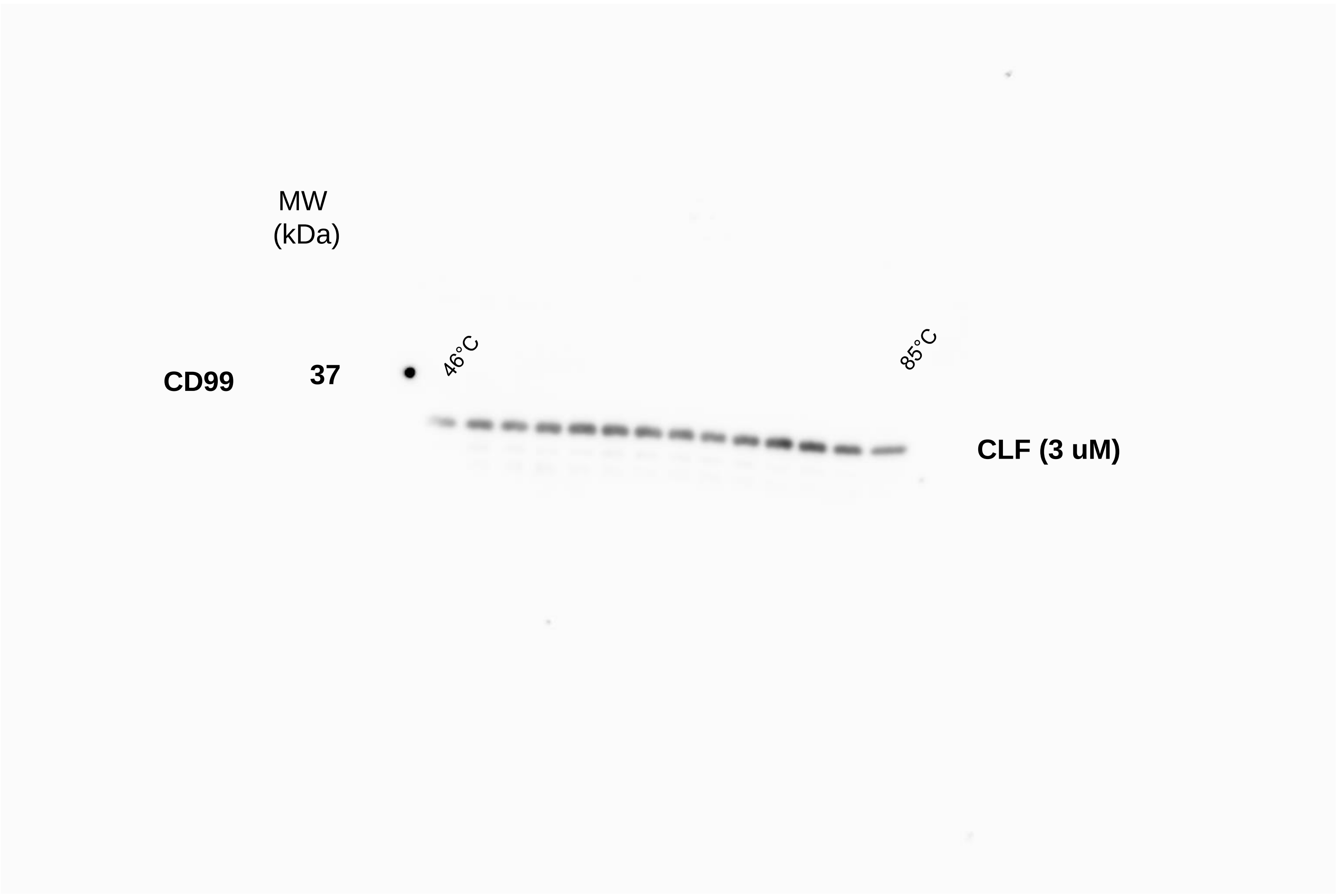

MW (kDa)
CD99
37
46°C
85°C
CLF (3 uM)

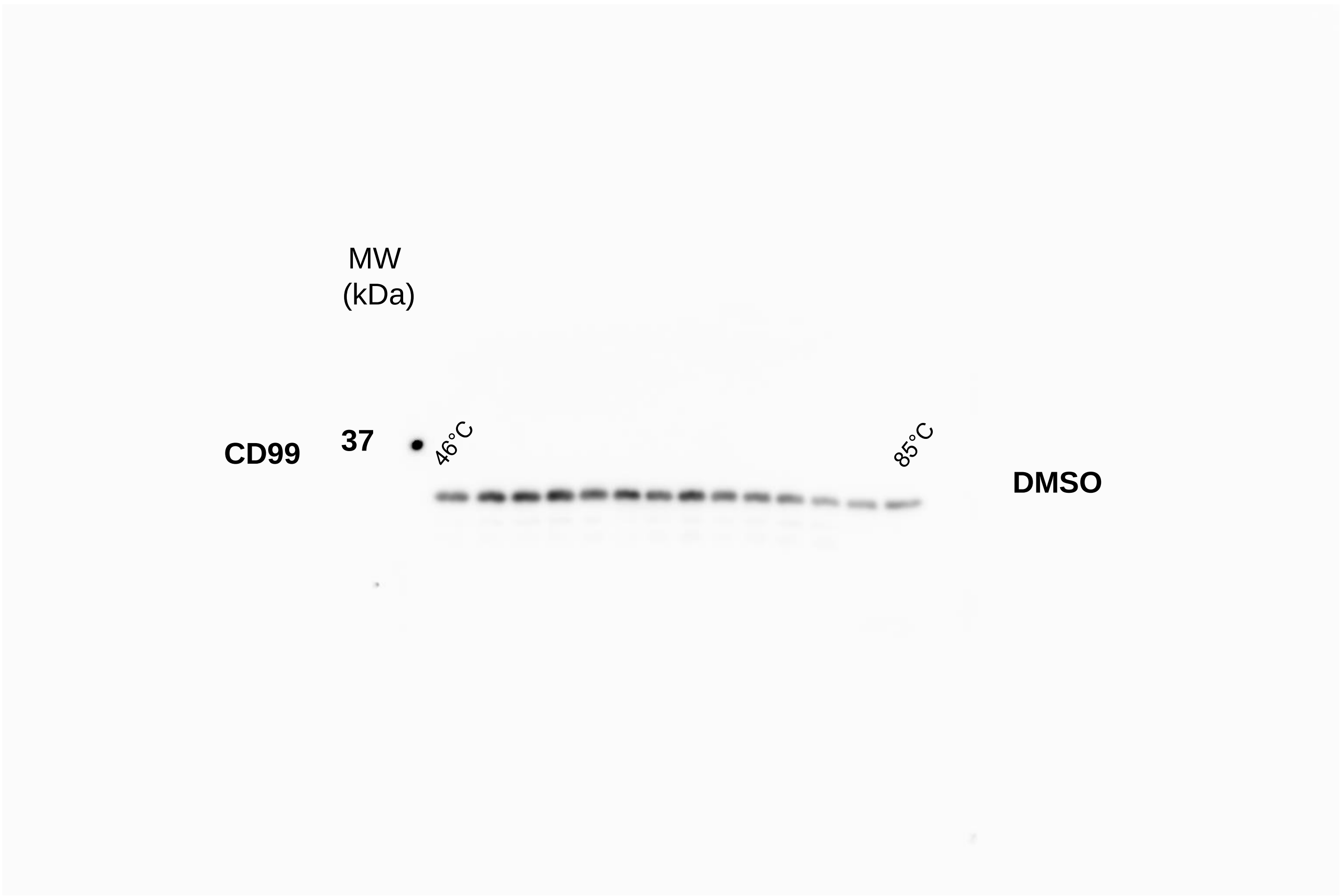

MW (kDa)
CD99
37
46°C
85°C
DMSO

Figure 1C, 1D

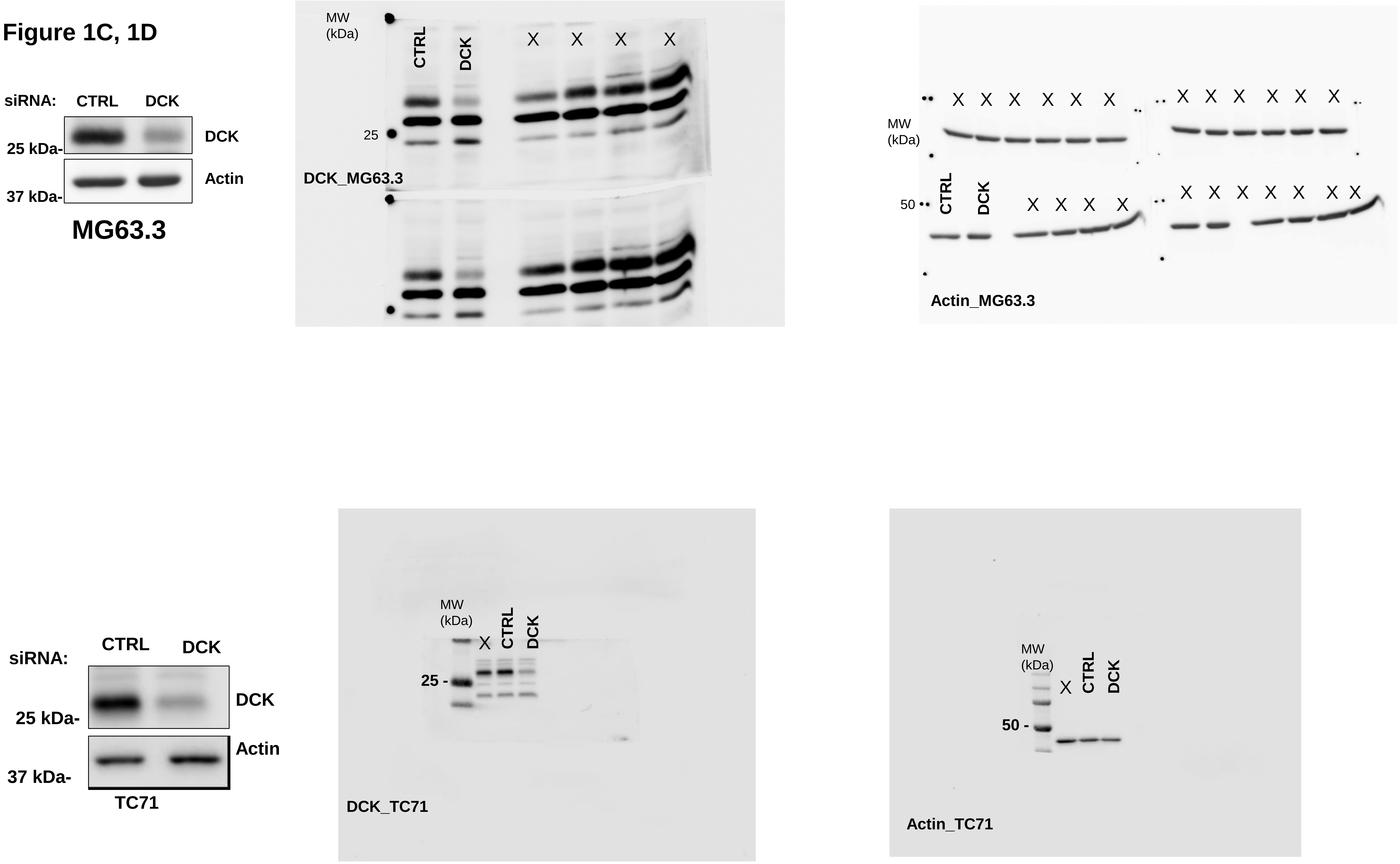

siRNA: CTRL DCK
25 kDa-
DCK
37 kDa-
Actin
MG63.3
MW (kDa)
CTRL
DCK
X X X X
25
DCK_MG63.3
X X X X X X
X X X X X X
MW (kDa)
50
CTRL
DCK
X X X X
X X X X X X X
Actin_MG63.3
siRNA: CTRL DCK
25 kDa-
DCK
Actin
37 kDa-
TC71
MW (kDa)
X
CTRL
DCK
25 -
DCK_TC71
MW (kDa)
X
CTRL
DCK
50 -
Actin_TC71

Figure 3A

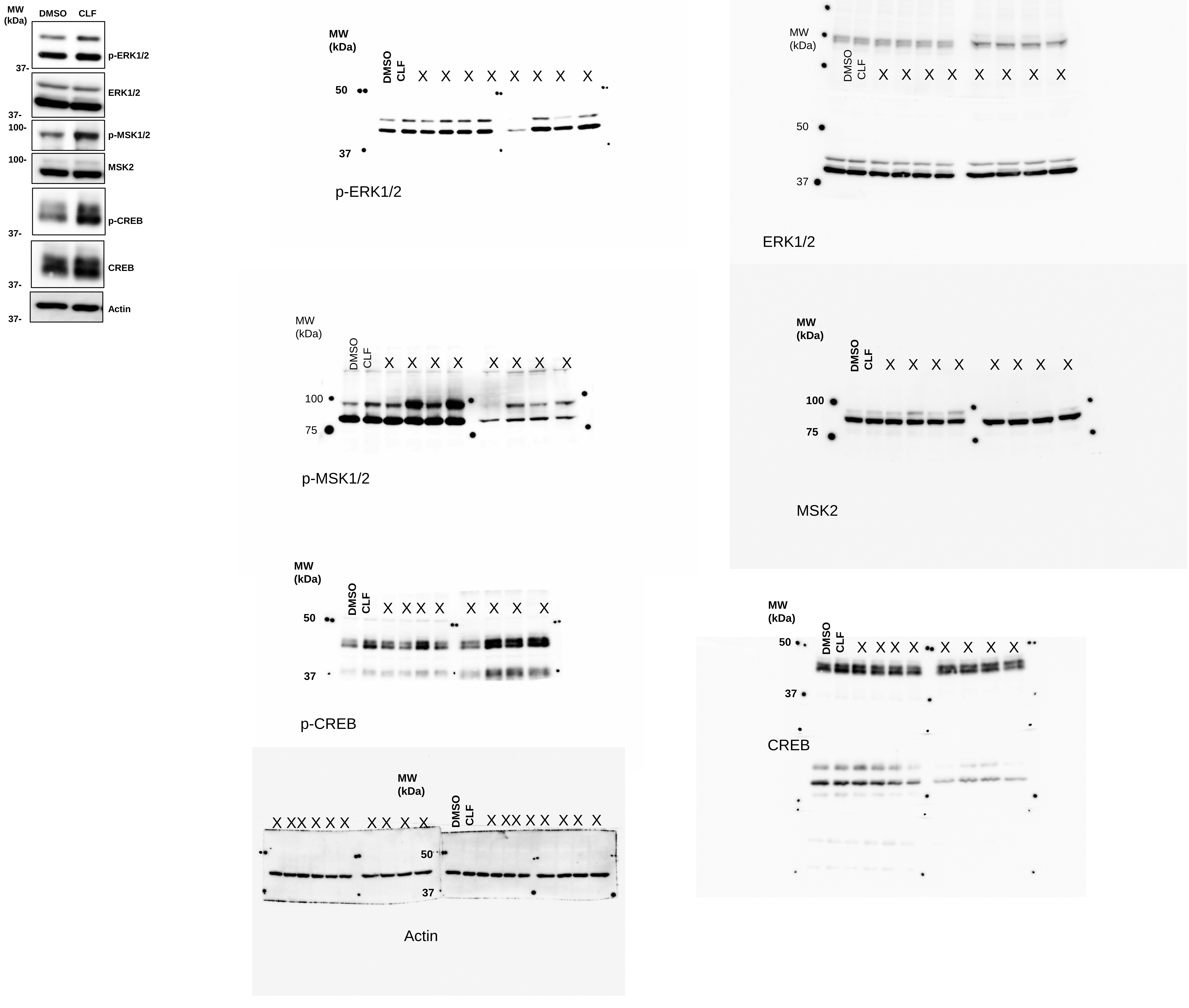
MW (kDa)
DMSO
CLF
p-ERK1/2
ERK1/2
p-MSK1/2
MSK2
p-CREB
CREB
Actin
37-
100-
50
37
100
75
X

Figure 3B

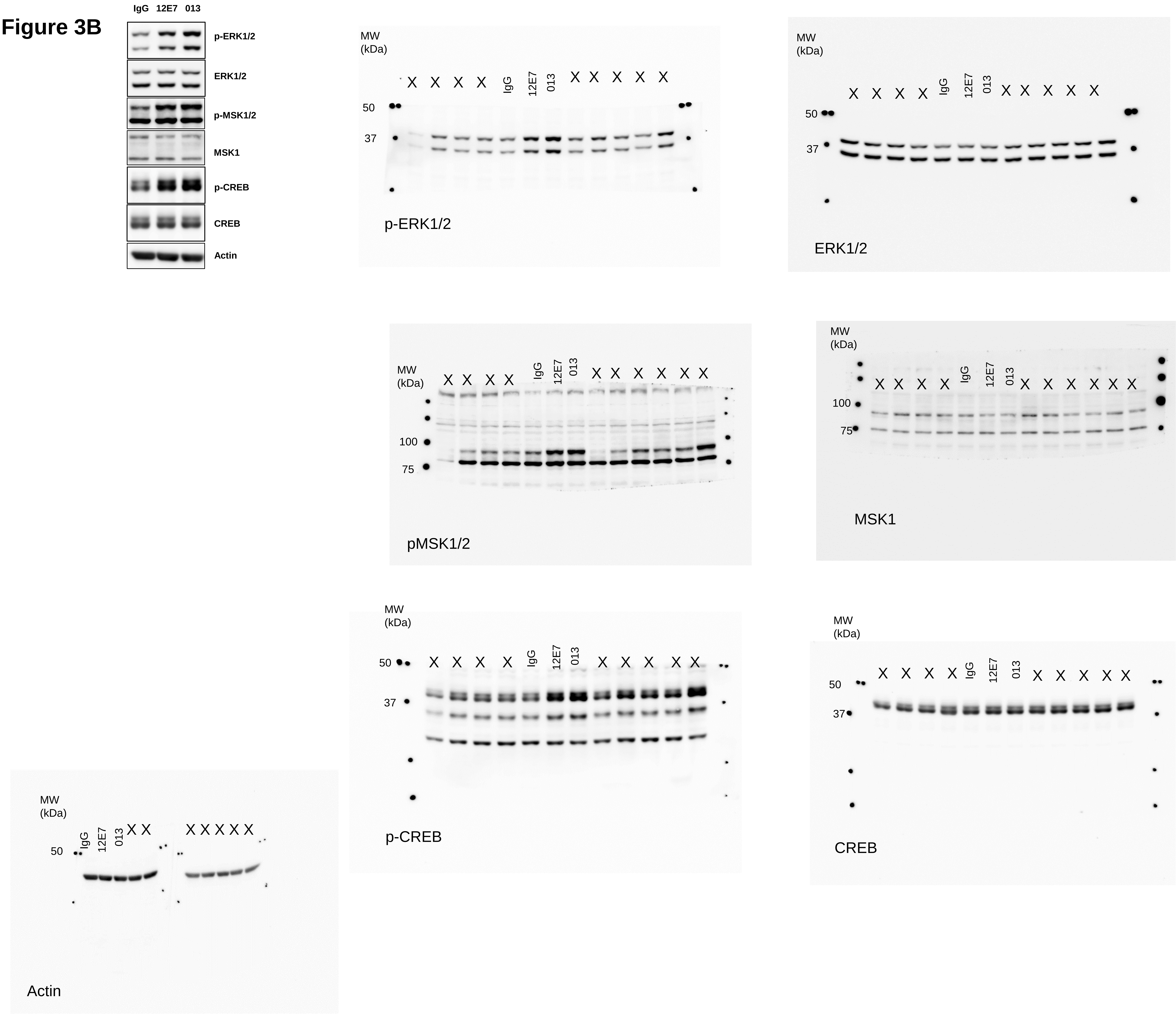

IgG 12E7 013
p-ERK1/2
ERK1/2
p-MSK1/2
MSK1
p-CREB
CREB
Actin
MW (kDa)
50
37
X X X X IgG 12E7 013 X X X X X
p-ERK1/2
MW (kDa)
50
37
X X X X IgG 12E7 013 X X X X X
ERK1/2
MW (kDa)
100
75
X X X X IgG 12E7 013 X X X X X X
pMSK1/2
MW (kDa)
100
75
X X X X IgG 12E7 013 X X X X X X
MSK1
MW (kDa)
50
37
X X X X IgG 12E7 013 X X X X X
p-CREB
MW (kDa)
50
37
X X X X IgG 12E7 013 X X X X X
CREB
MW (kDa)
50
IgG 12E7 013 X X X X X X X
Actin

Figure 3C

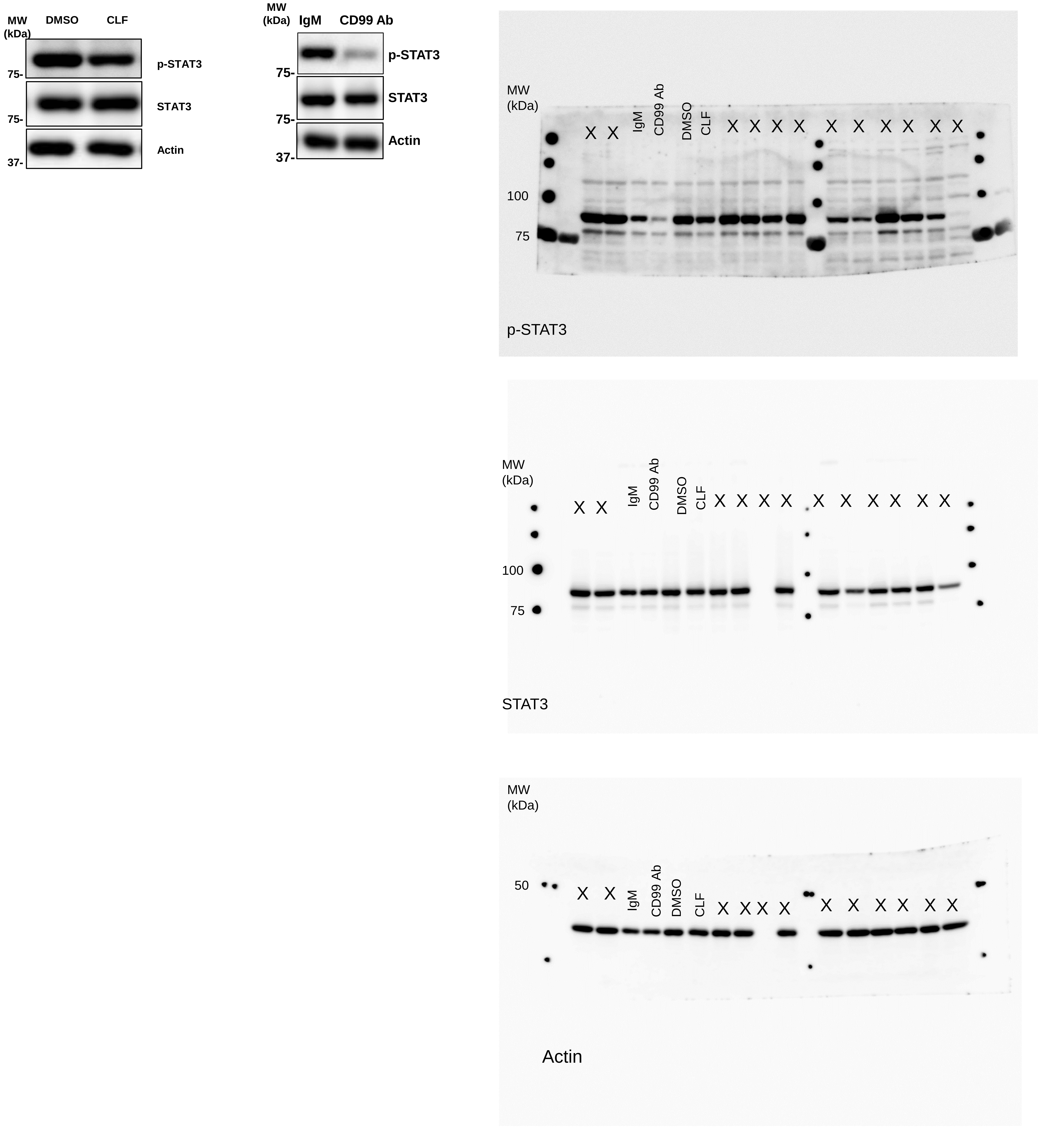

MW (kDa)
DMSO
CLF
75-
75-
37-
p-STAT3
STAT3
Actin
MW (kDa)
IgM
CD99 Ab
75-
75-
37-
p-STAT3
STAT3
Actin
MW (kDa)
IgM
CD99 Ab
DMSO
CLF
X
100
75
p-STAT3
MW (kDa)
IgM
CD99 Ab
DMSO
CLF
X
100
75
STAT3
MW (kDa)
50
IgM
CD99 Ab
DMSO
CLF
X
Actin

Figure 3D

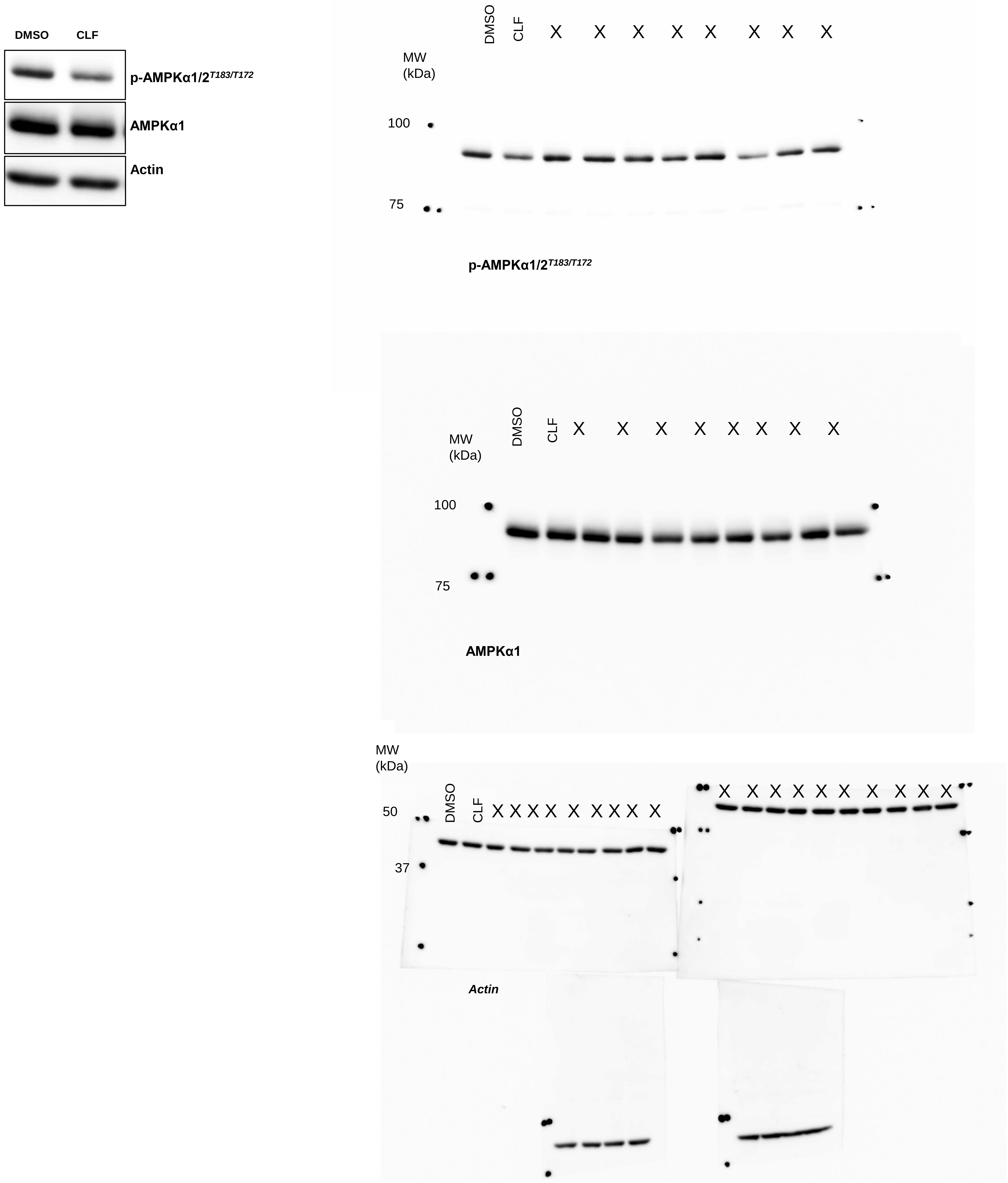
DMSO
CLF
p-AMPKα1/2T183/T172
AMPKα1
Actin
DMSO
CLF
X X X X X X X X
MW
(kDa)
100
75
p-AMPKα1/2T183/T172
DMSO
CLF
X X X X X X X X
MW
(kDa)
100
75
AMPKα1
MW
(kDa)
50
37
DMSO
CLF
X X X X X X X X X
X X X X X X X X X X
Actin

Figure 3E

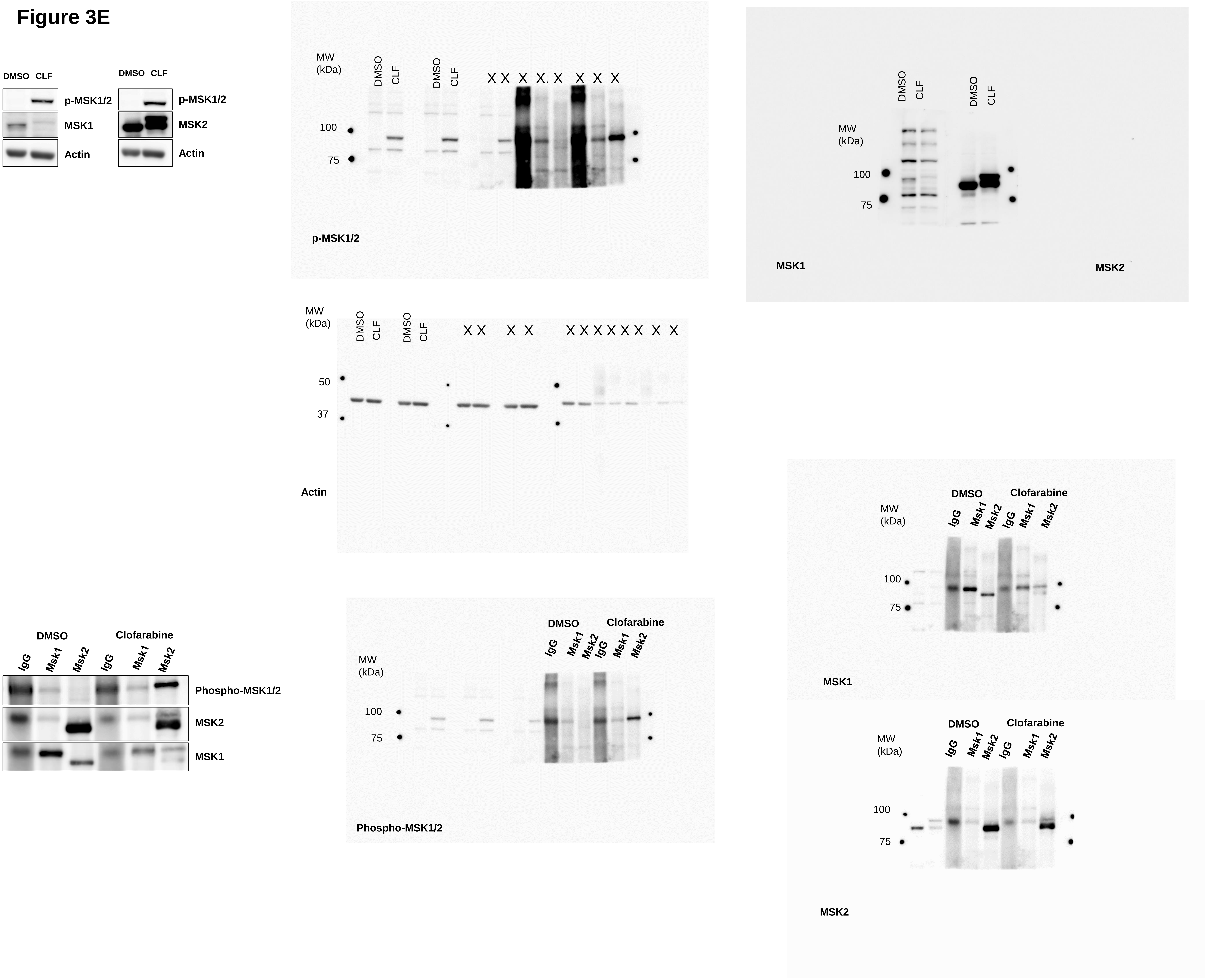
DMSO
CLF
p-MSK1/2
MSK1
Actin
DMSO
CLF
p-MSK1/2
MSK2
Actin
MW (kDa)
DMSO
CLF
DMSO
CLF
X X X X. X X X X
100
75
p-MSK1/2
DMSO
CLF
DMSO
CLF
MW (kDa)
100
75
MSK1
MSK2
MW (kDa)
DMSO
CLF
DMSO
CLF
X X X X X X X X X X X X
50
37
Actin
DMSO
Clofarabine
IgG
Msk1
Msk2
IgG
Msk1
Msk2
MW (kDa)
100
75
MSK1
DMSO
Clofarabine
IgG
Msk1
Msk2
IgG
Msk1
Msk2
Phospho-MSK1/2
MSK2
MSK1
MW (kDa)
DMSO
Clofarabine
IgG
Msk1
Msk2
IgG
Msk1
Msk2
100
75
Phospho-MSK1/2
DMSO
Clofarabine
IgG
Msk1
Msk2
IgG
Msk1
Msk2
MW (kDa)
100
75
MSK2

Figure 4A

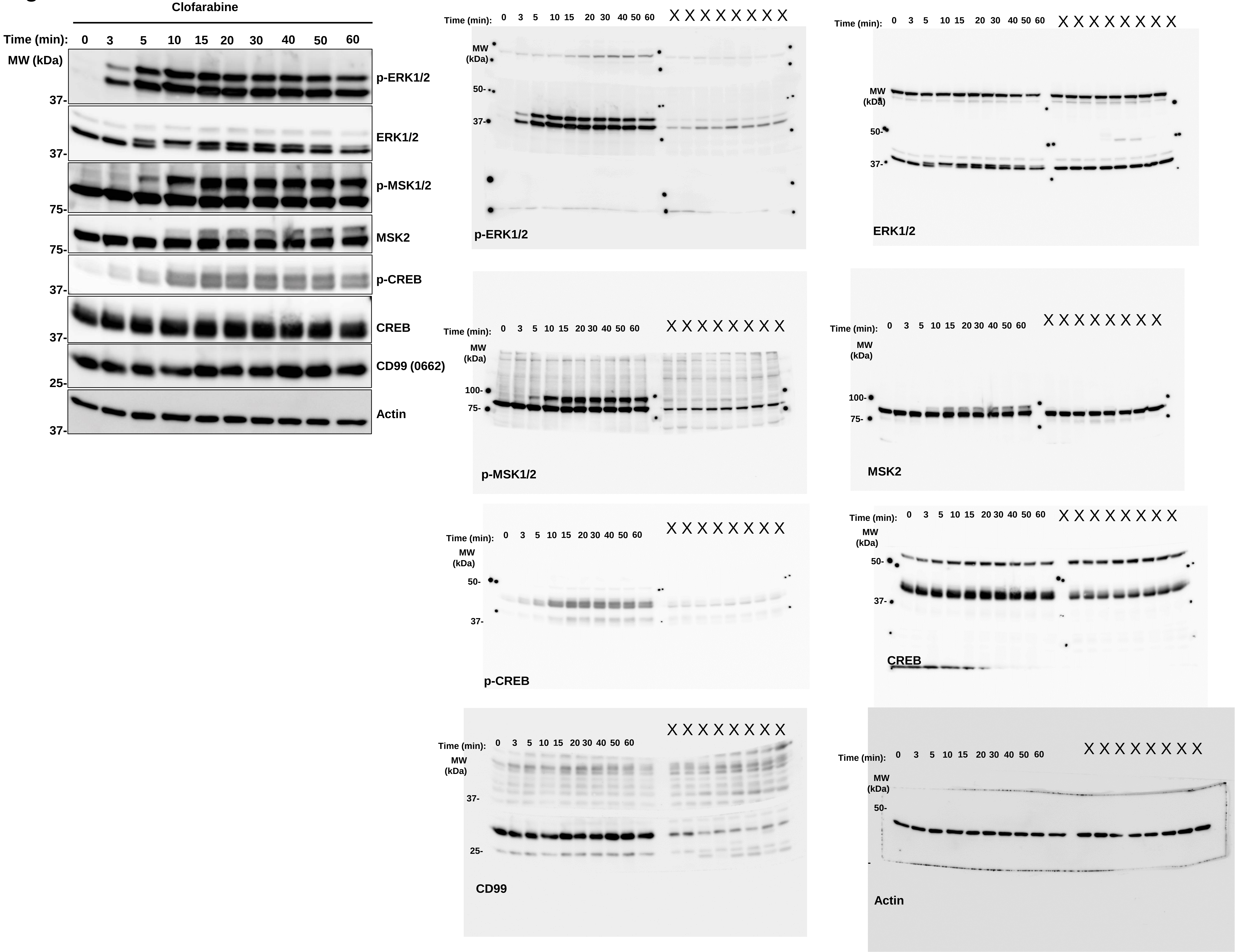

Clofarabine
Time (min): 0 3 5 10 15 20 30 40 50 60
MW (kDa)
p-ERK1/2
ERK1/2
p-MSK1/2
MSK2
p-CREB
CREB
CD99 (0662)
Actin
37-
75-
25-
50-
100-
X X X X X X X X
MW (kDa)
p-ERK1/2
ERK1/2
p-MSK1/2
MSK2
p-CREB
CREB
CD99
Actin

Figure 4B

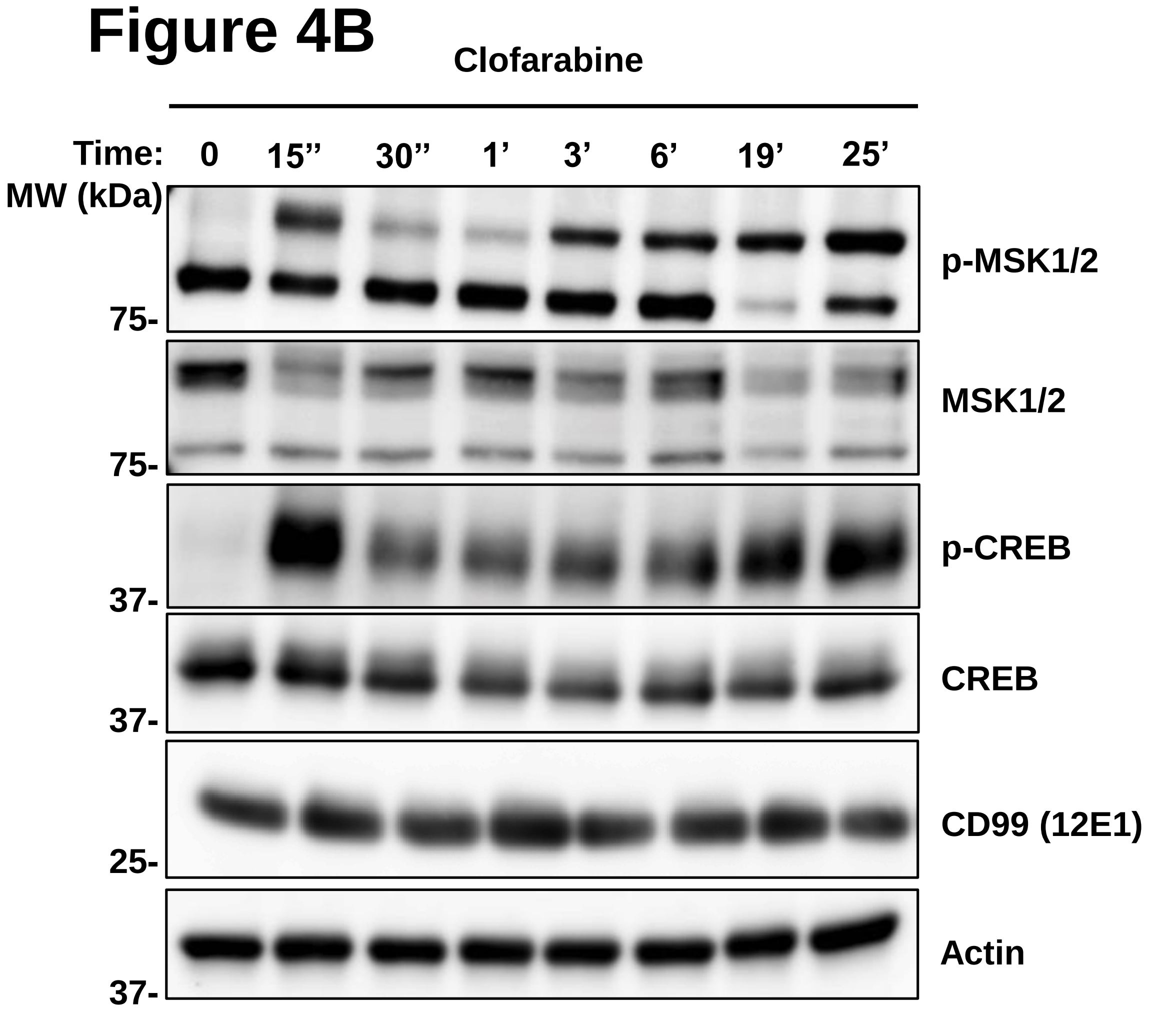
Clofarabine
Time: 0 15'' 30'' 1' 3' 6' 19' 25'
MW (kDa)
75-
p-MSK1/2
75-
MSK1/2
37-
p-CREB
37-
CREB
25-
CD99 (12E1)
37-
Actin

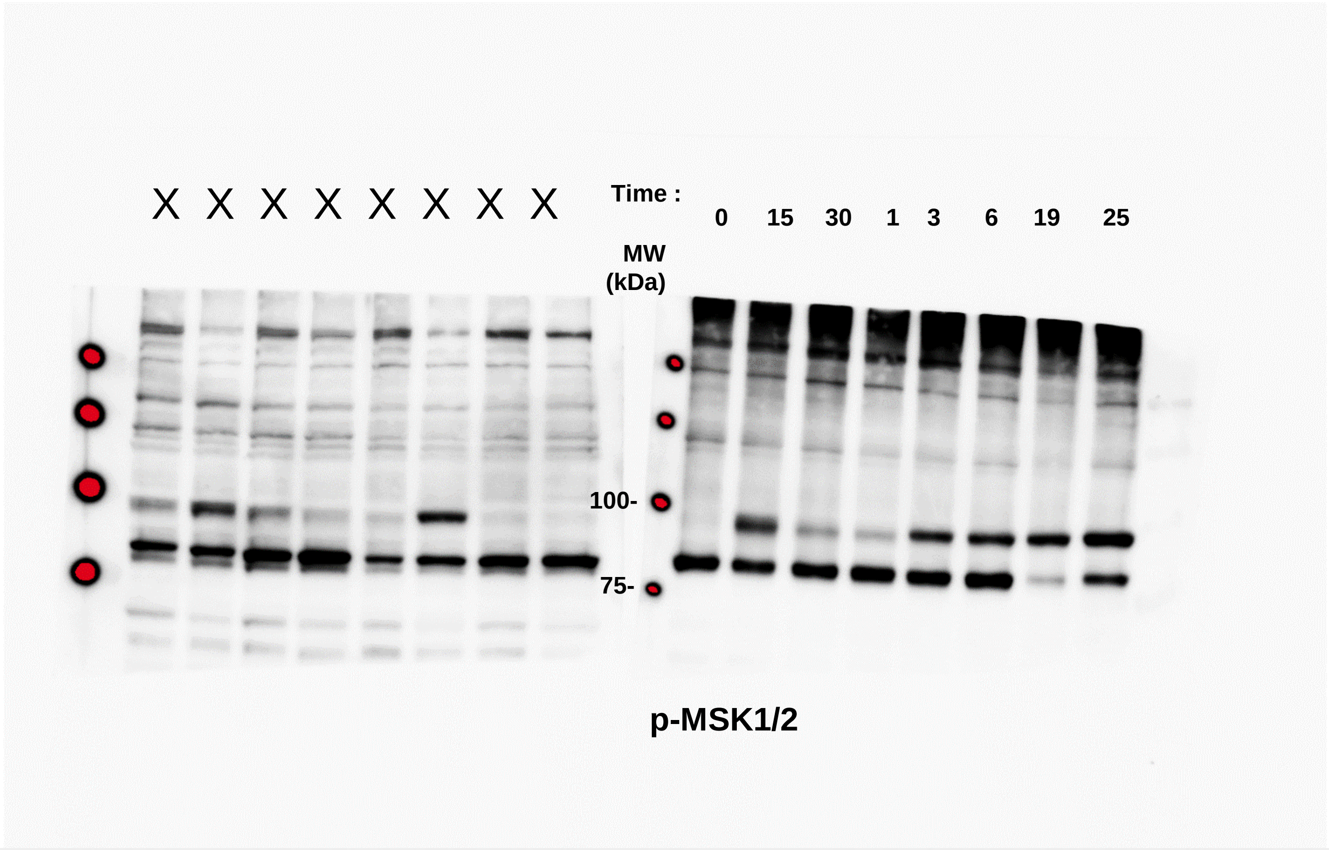
X X X X X X X X
Time : 0 15 30 1 3 6 19 25
MW (kDa)
100-
75-
p-MSK1/2

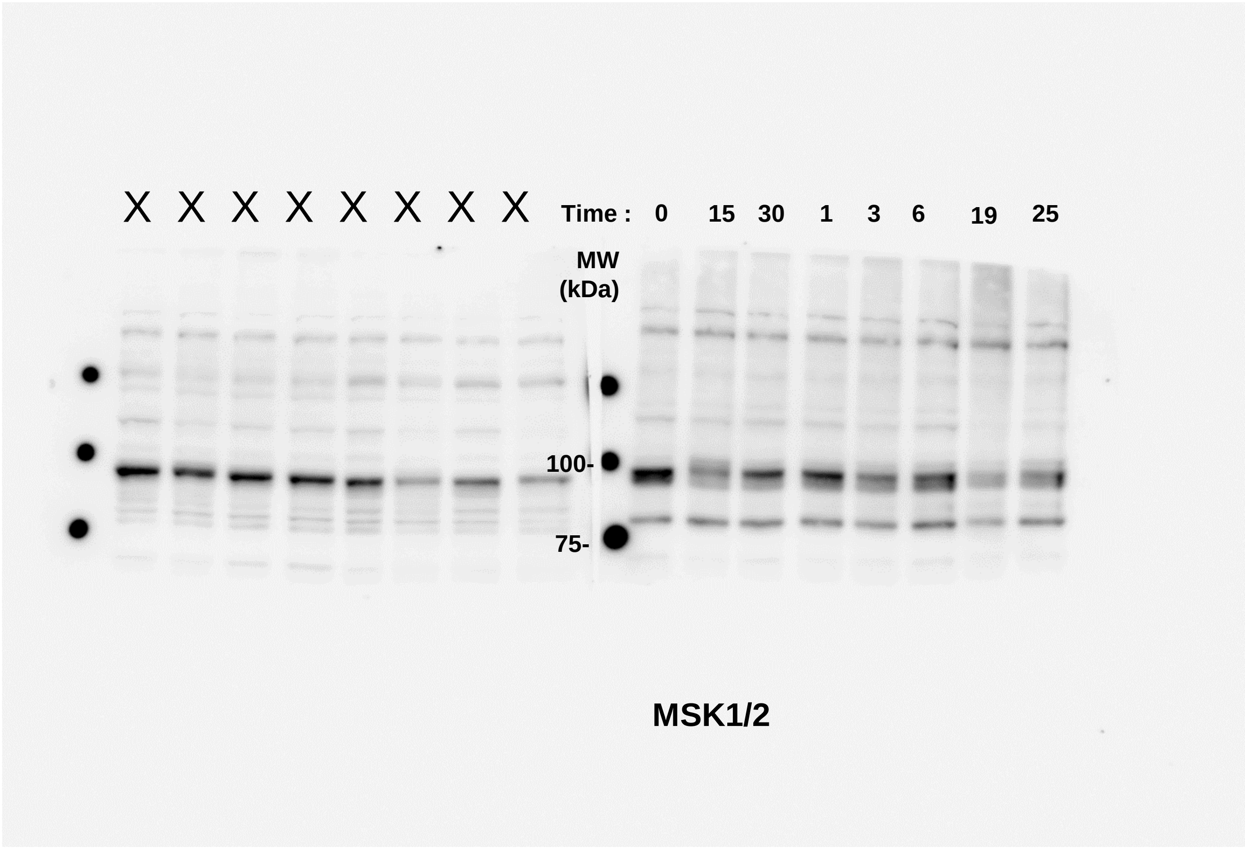
X X X X X X X X
Time : 0 15 30 1 3 6 19 25
MW (kDa)
100-
75-
MSK1/2

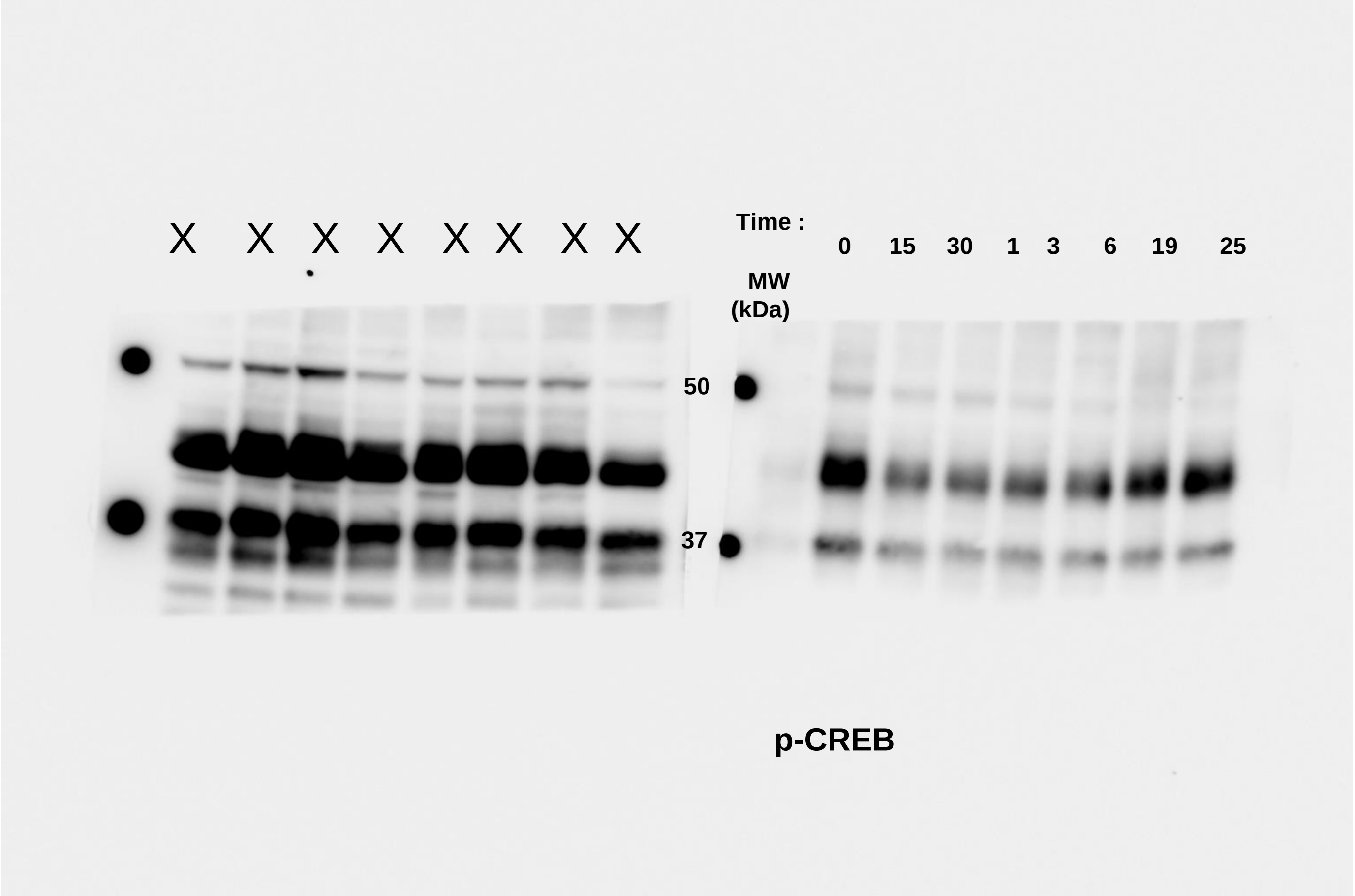
X X X X X X X X
Time : 0 15 30 1 3 6 19 25
MW (kDa)
50
37
p-CREB

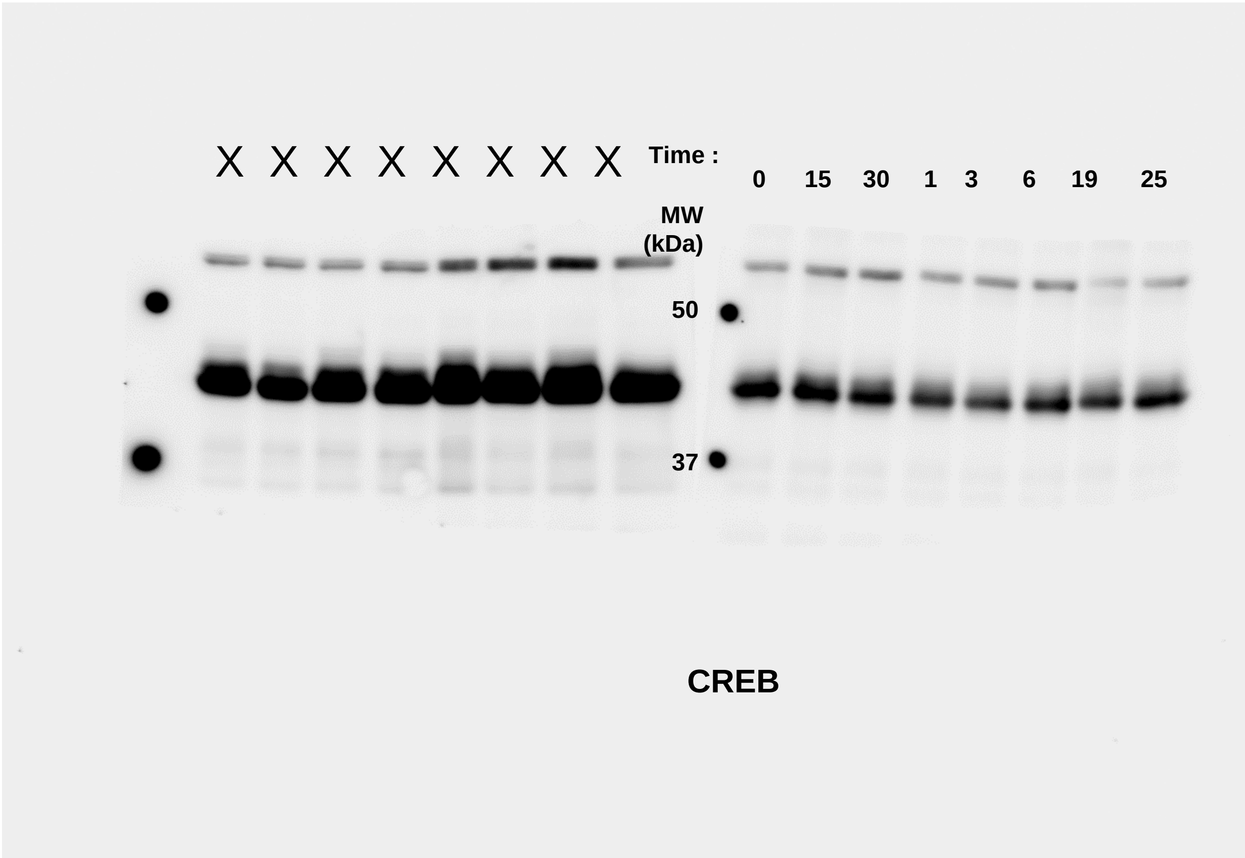
X X X X X X X X
Time : 0 15 30 1 3 6 19 25
MW (kDa)
50
37
CREB

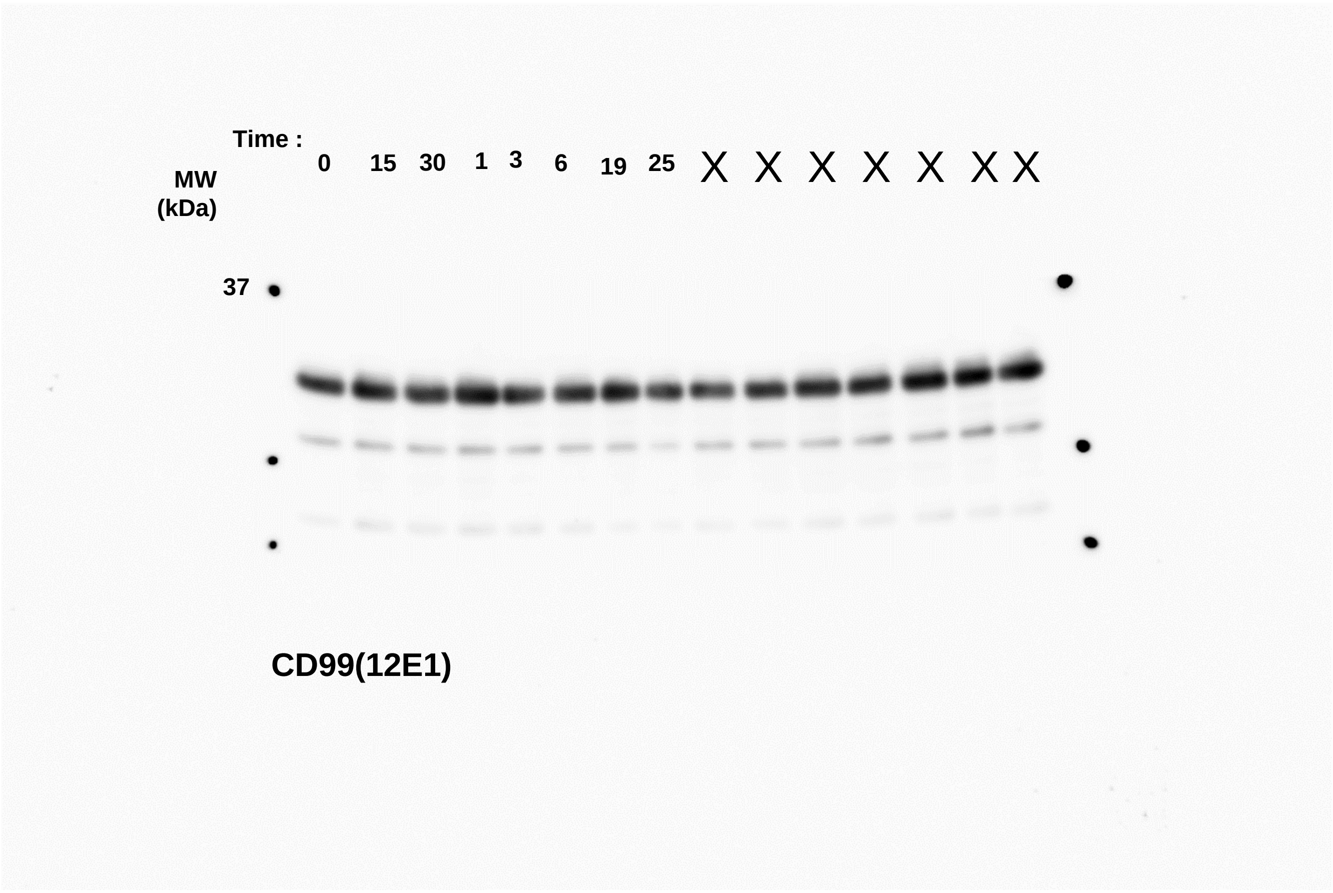
Time : 0 15 30 1 3 6 19 25 X X X X X X X
MW (kDa)
37
CD99(12E1)

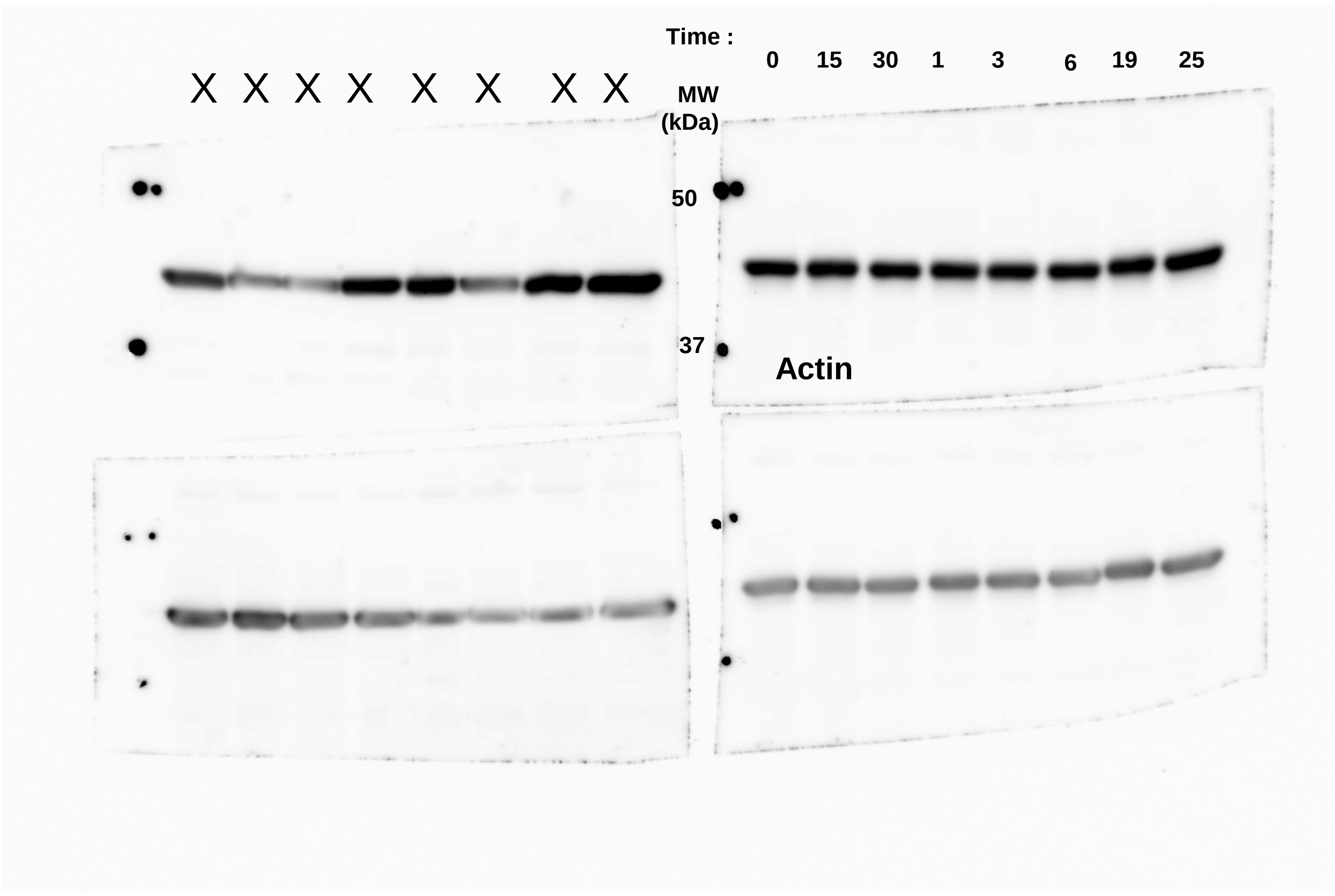
X X X X X X X X
Time : 0 15 30 1 3 6 19 25
MW (kDa)
50
37
Actin

Figure 5A; STA-ET-7.2

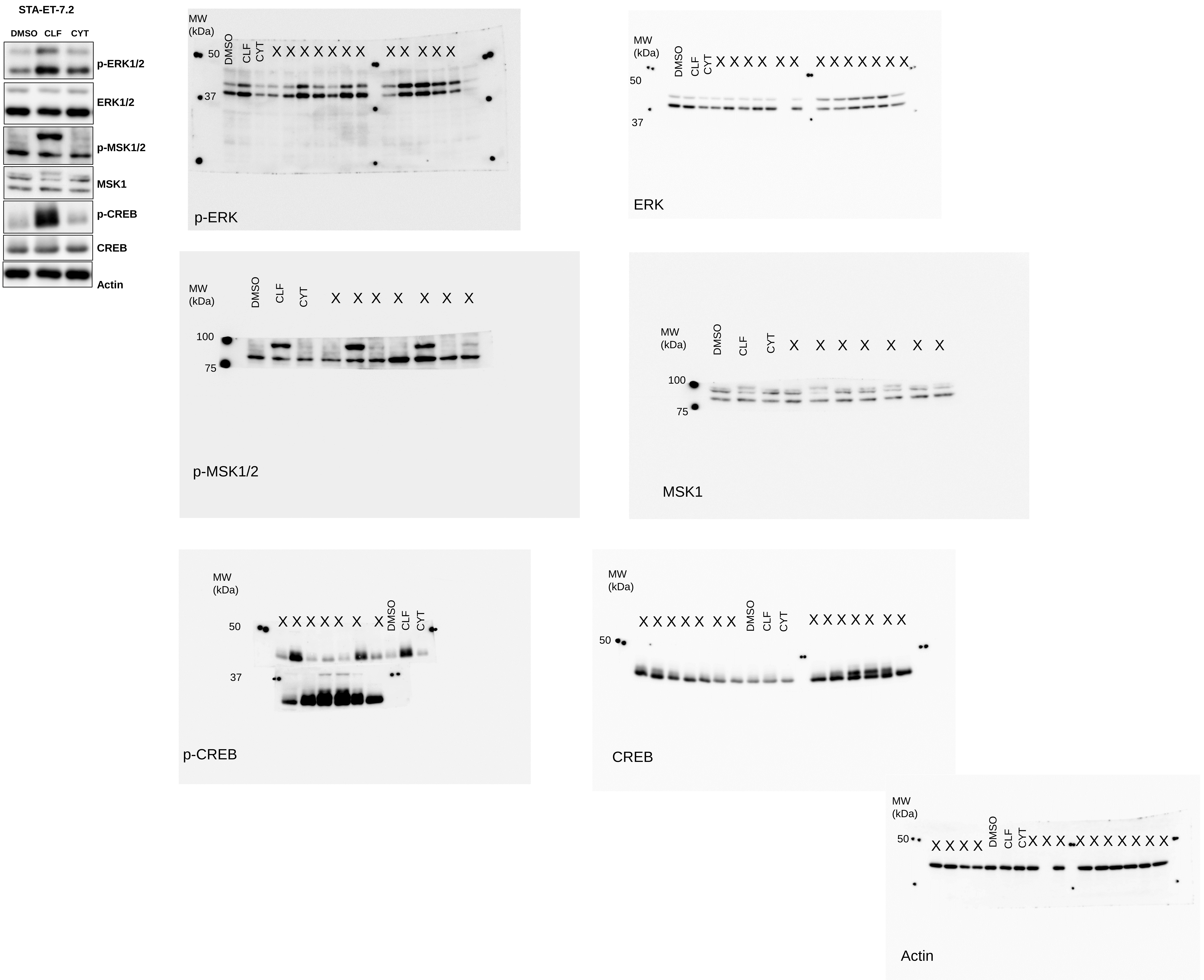

Figure 5B
RD-ES and A4573

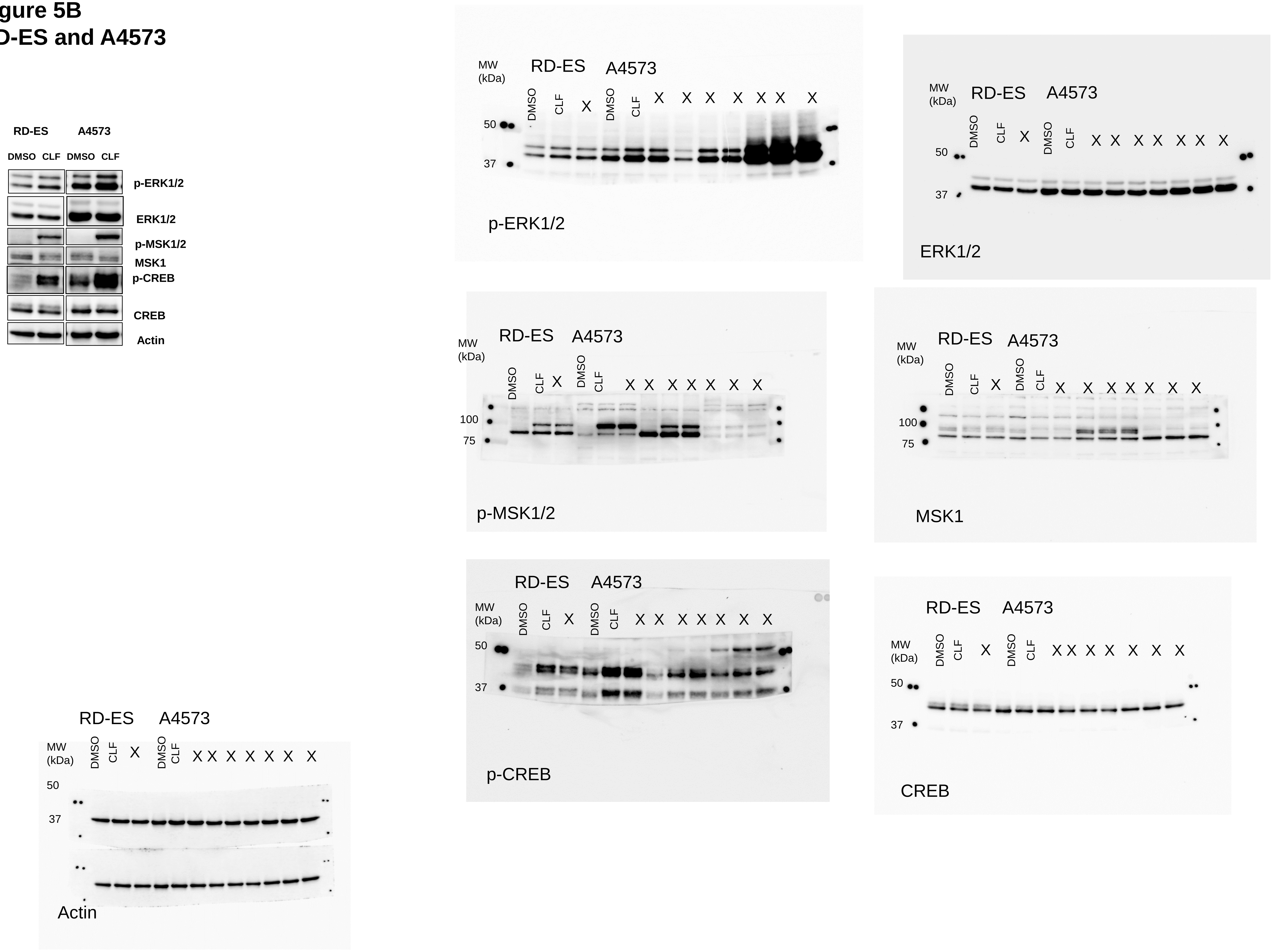

Figure 5B
STA-ET-7.2

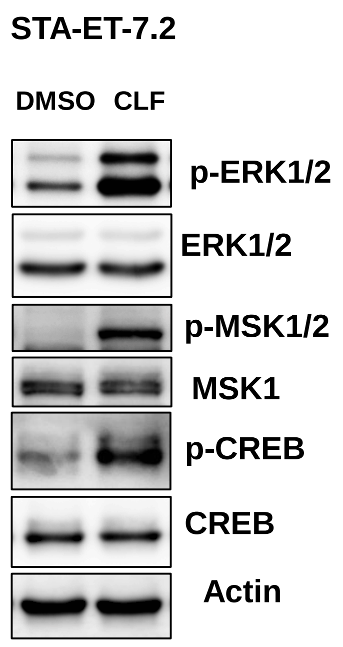

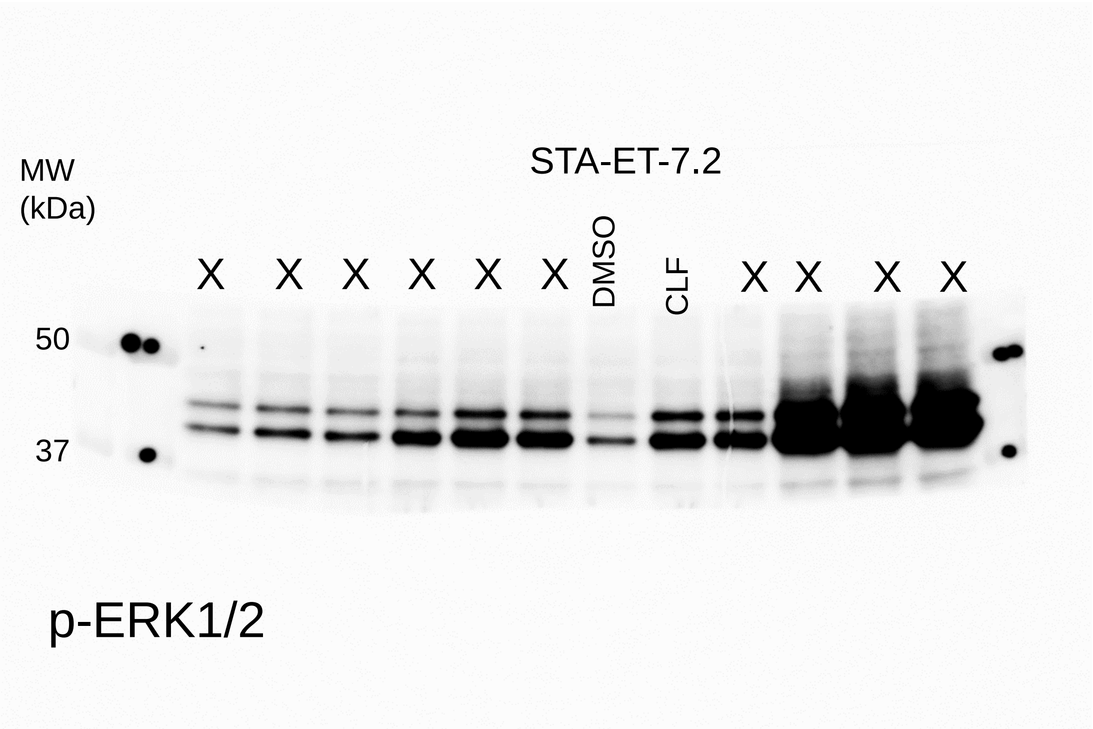

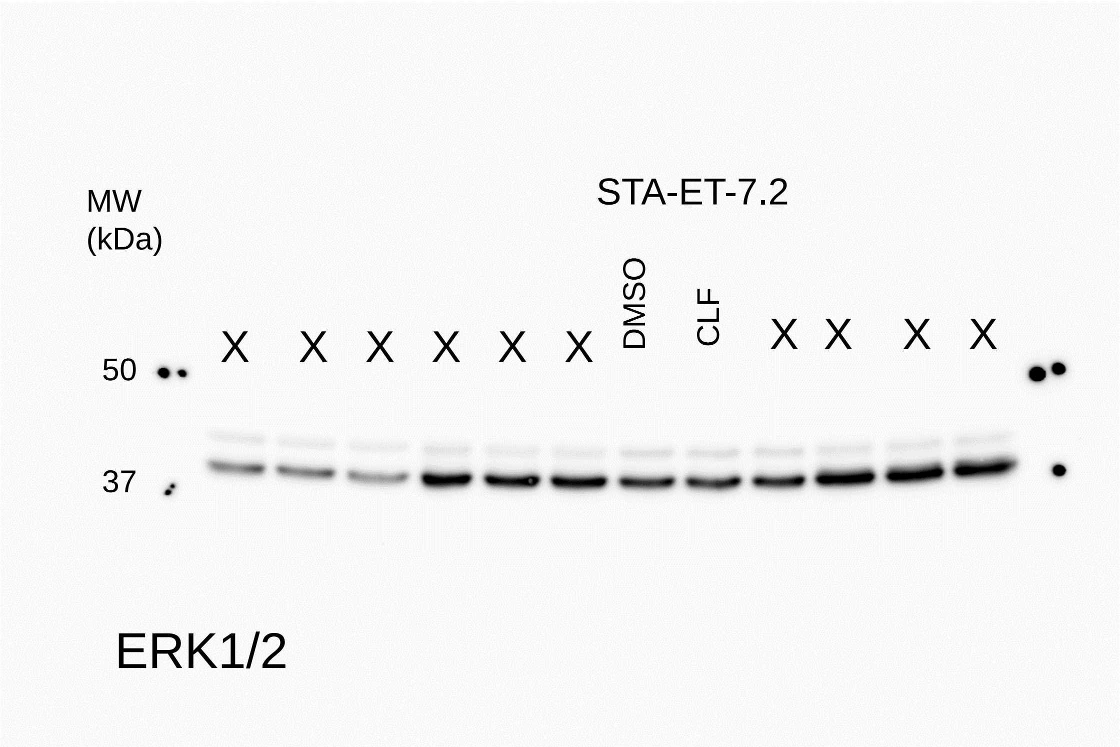

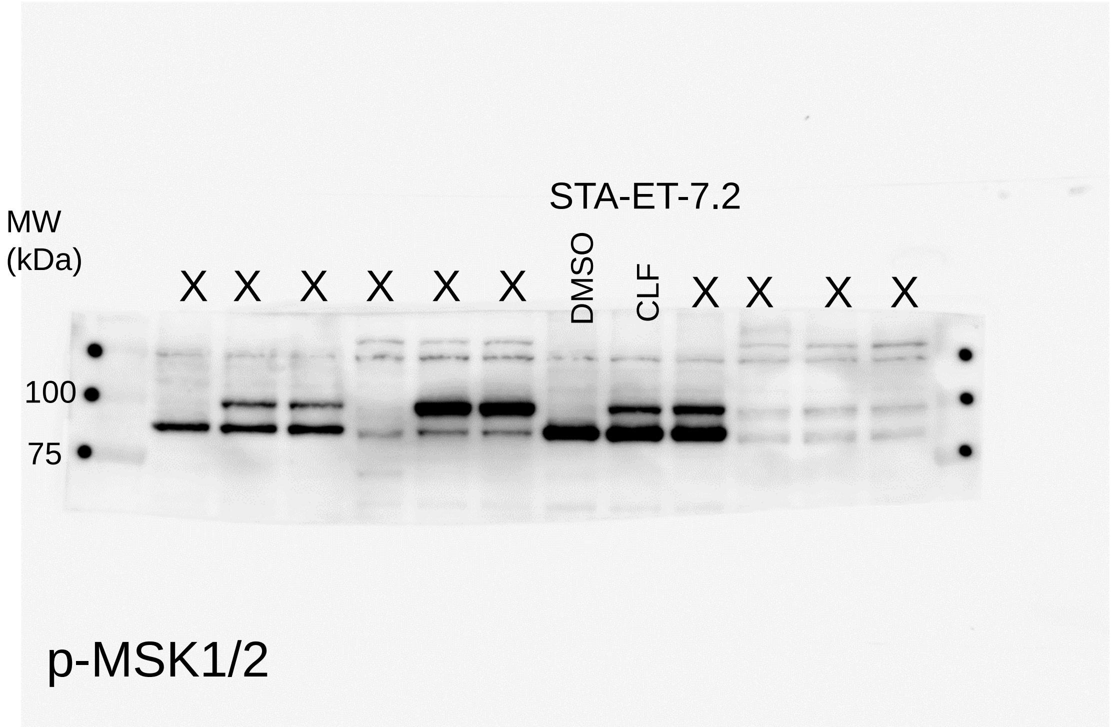

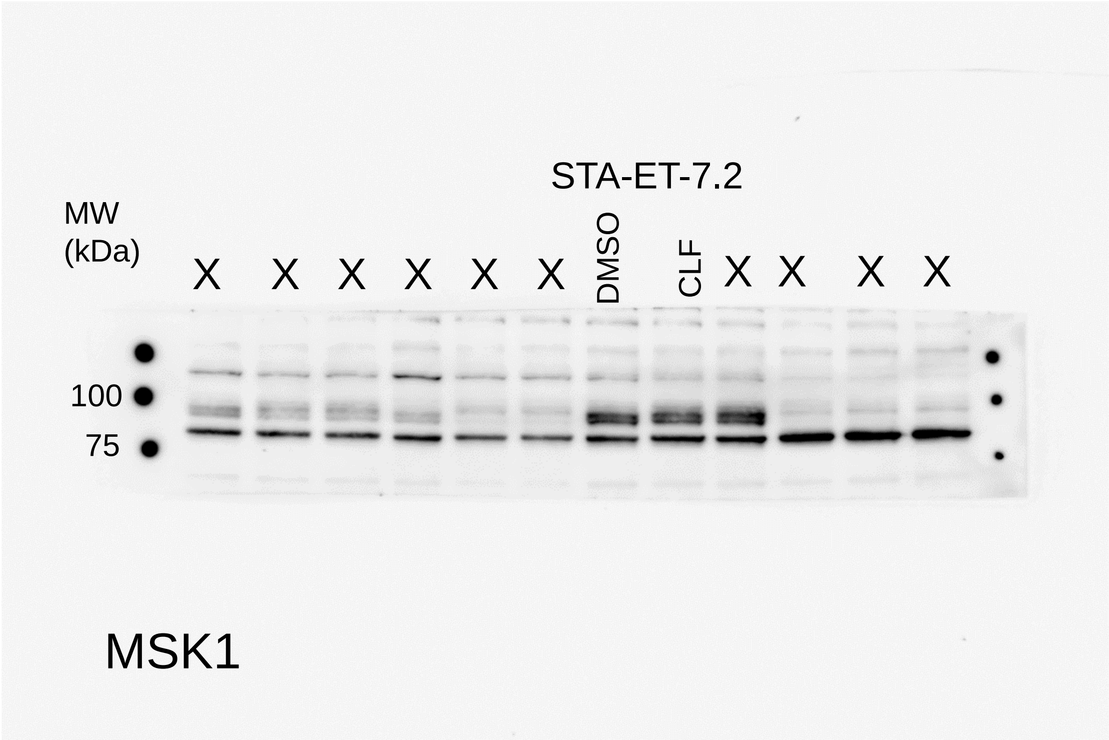

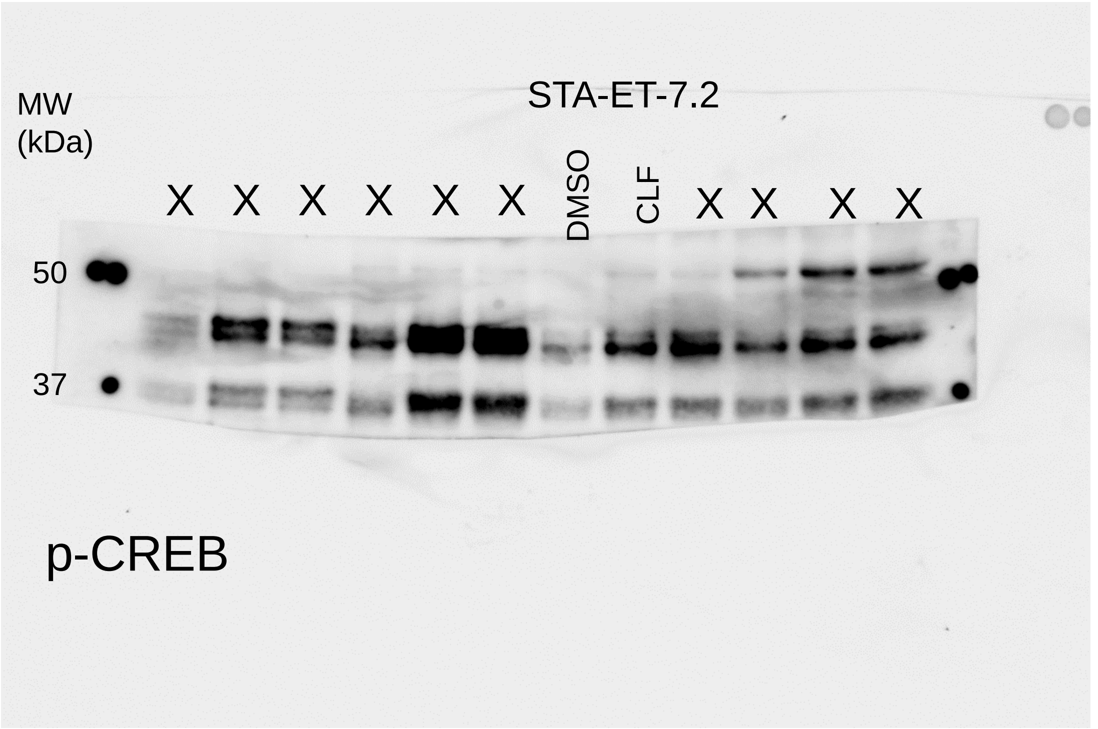

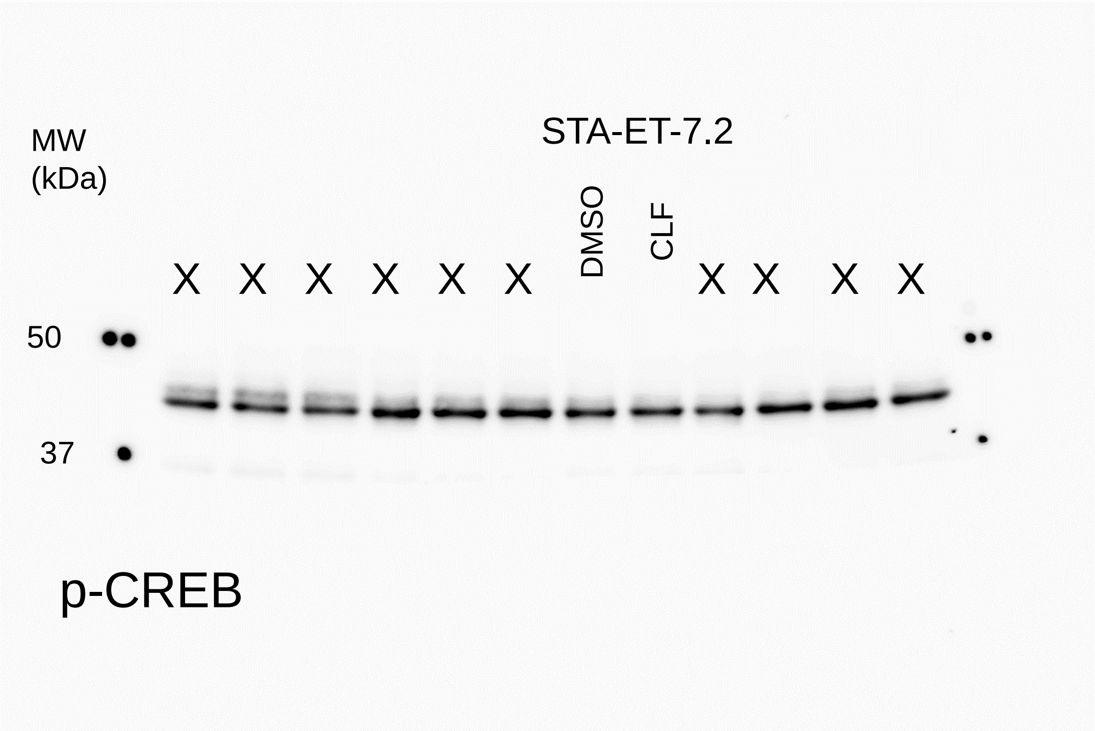

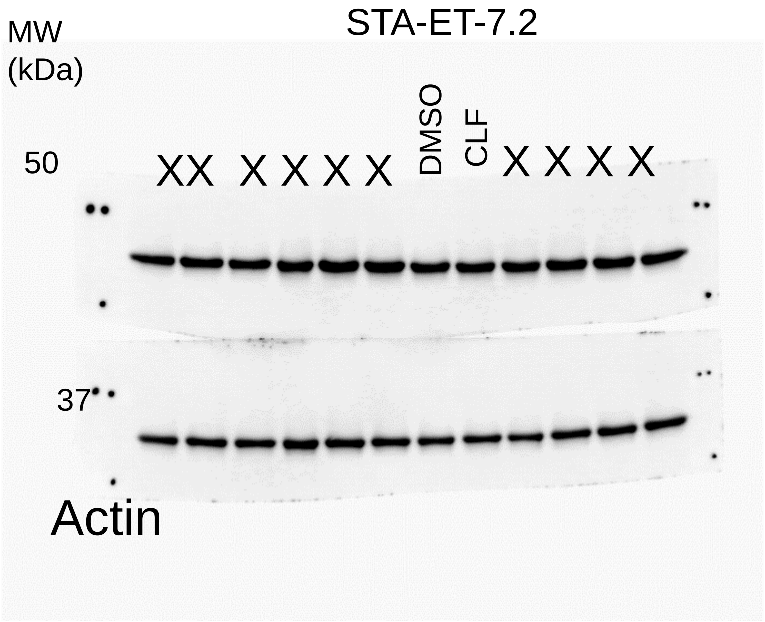

Figure 5B
TC32

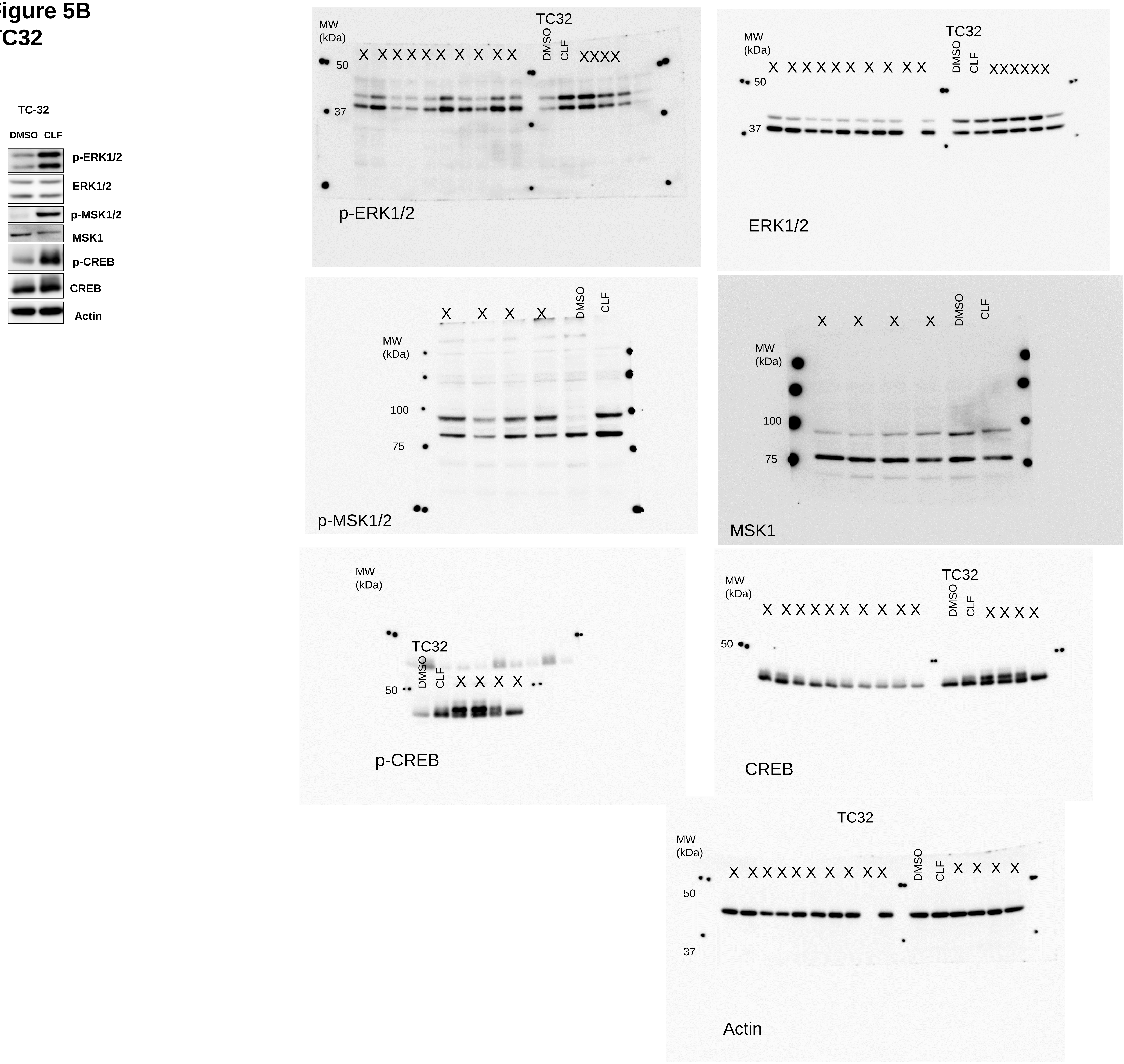

Figure 5B
TC71

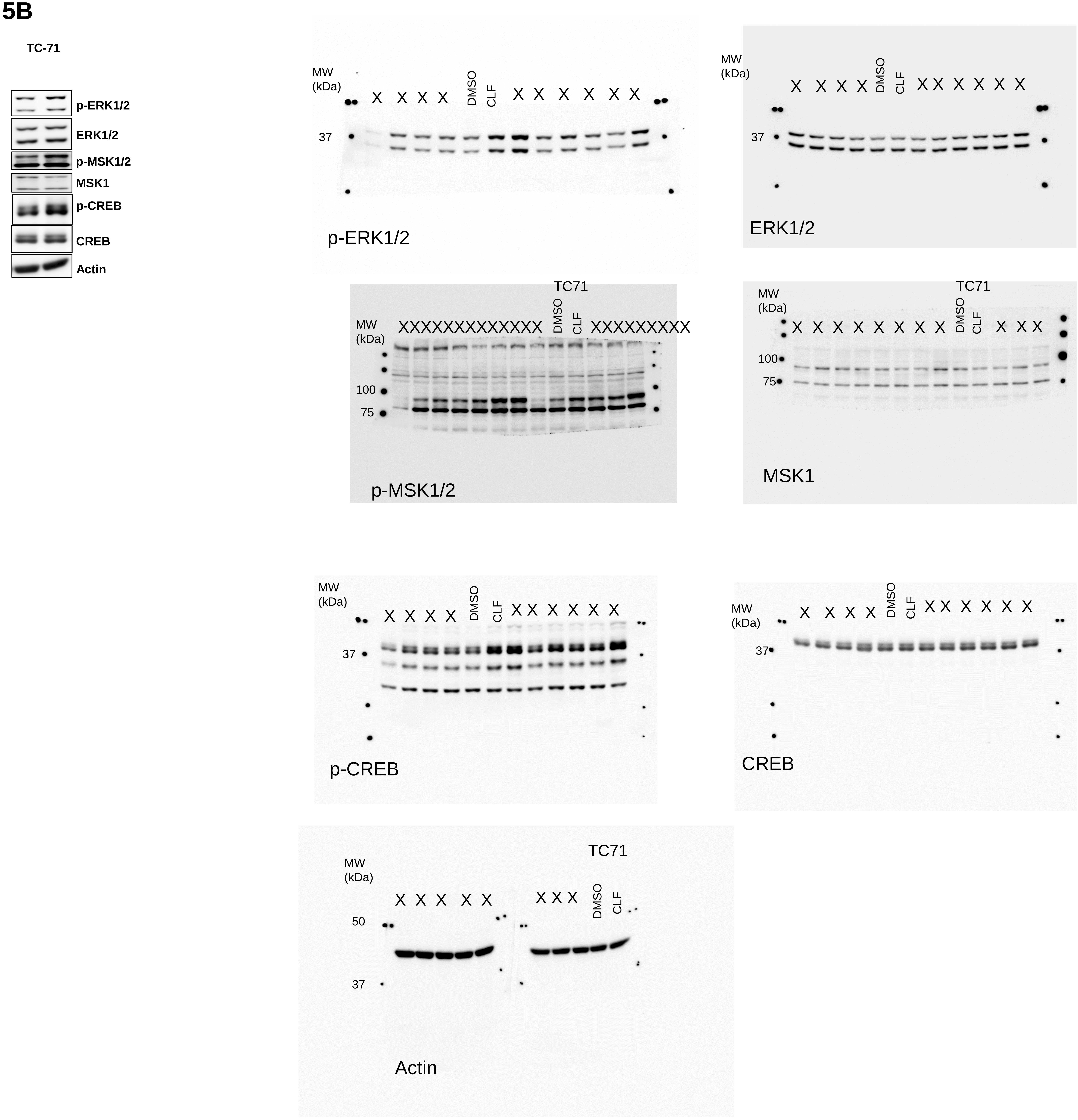

Figure 5C

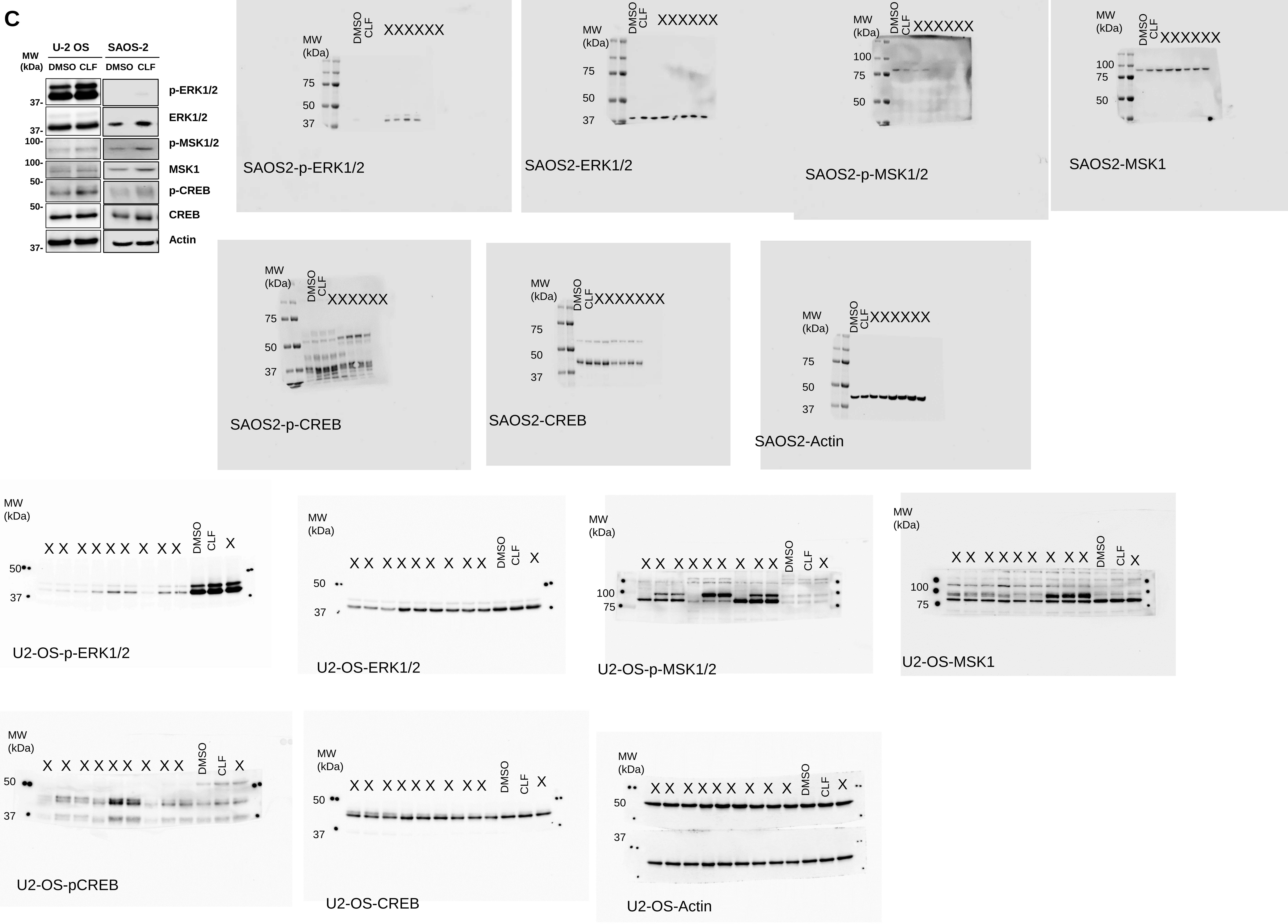

C
U-2 OS
SAOS-2
MW (kDa)
DMSO CLF DMSO CLF
p-ERK1/2
ERK1/2
p-MSK1/2
MSK1
p-CREB
CREB
Actin
37-
37-
100-
100-
50-
50-
37-
SAOS2-p-ERK1/2
SAOS2-ERK1/2
SAOS2-p-MSK1/2
SAOS2-MSK1
SAOS2-p-CREB
SAOS2-CREB
SAOS2-Actin
U2-OS-p-ERK1/2
U2-OS-ERK1/2
U2-OS-p-MSK1/2
U2-OS-MSK1
U2-OS-pCREB
U2-OS-CREB
U2-OS-Actin

Figure S1

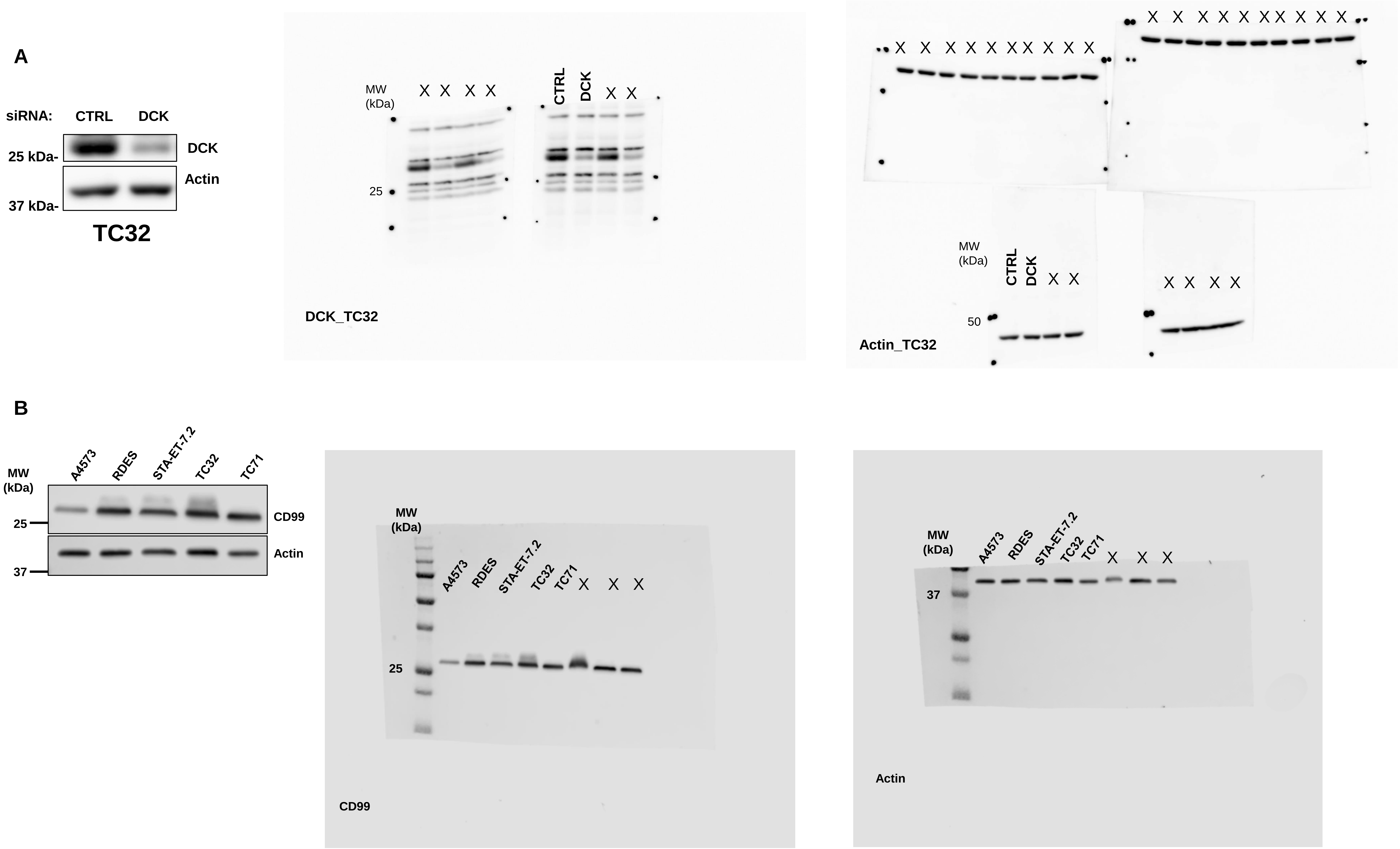

A
siRNA: CTRL DCK
25 kDa-
DCK
Actin
37 kDa-
TC32
MW (kDa)
X X X X
CTRL
DCK
X X
25
DCK_TC32
X X X X X X X X X X
X X X X X X X X X X
MW (kDa)
CTRL
DCK
X X
X X X X
50
Actin_TC32
B
MW (kDa)
A4573
RDES
STA-ET-7.2
TC32
TC71
25
37
CD99
Actin
MW (kDa)
A4573
RDES
STA-ET-7.2
TC32
TC71
X X X
25
CD99
MW (kDa)
A4573
RDES
STA-ET-7.2
TC32
TC71
X X X
37
Actin

Sup Fıgure3A

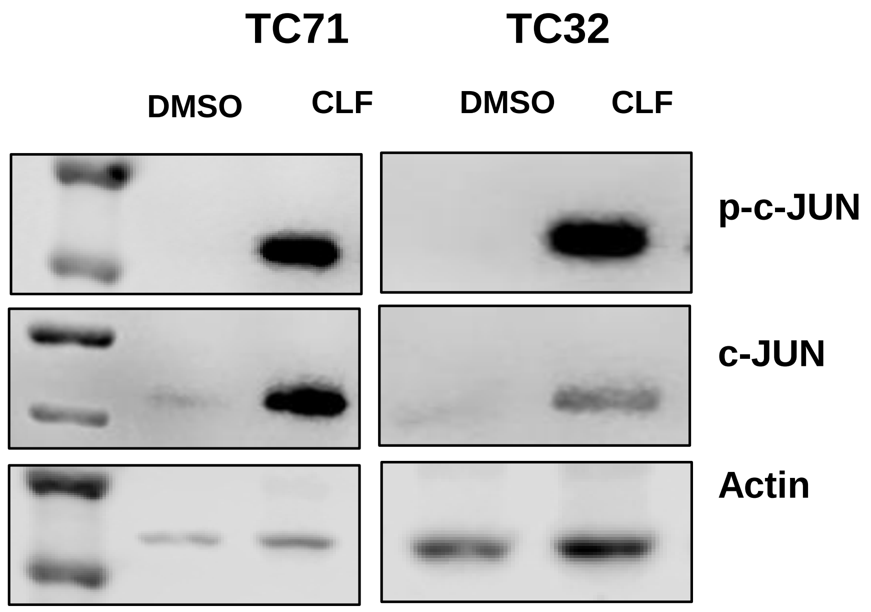

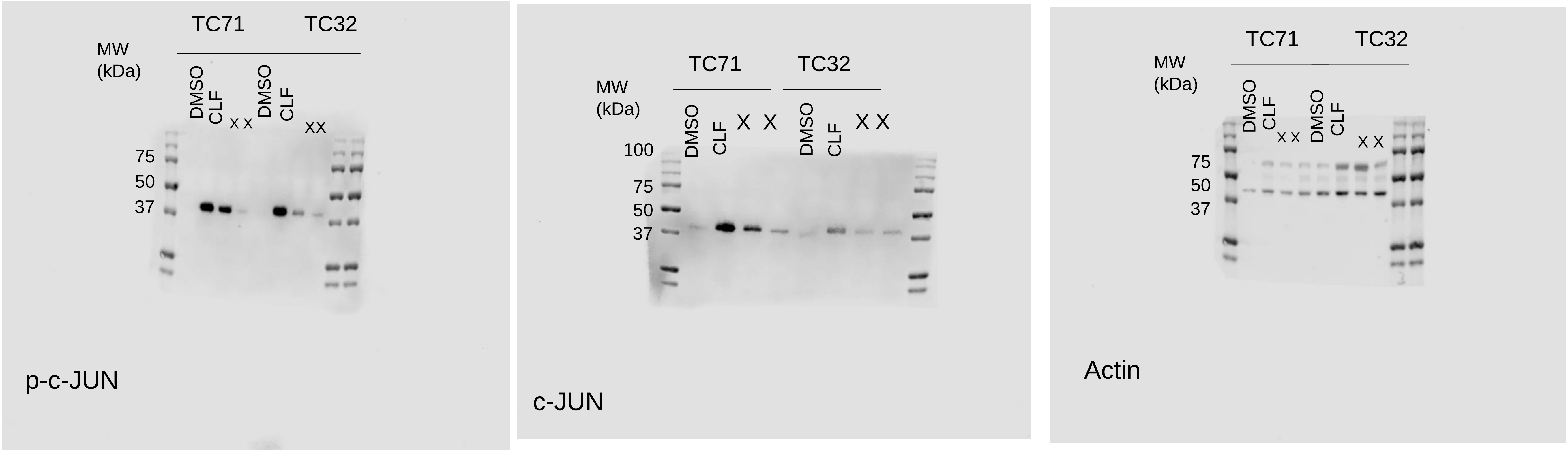

Sup Fıgure3B

A4573

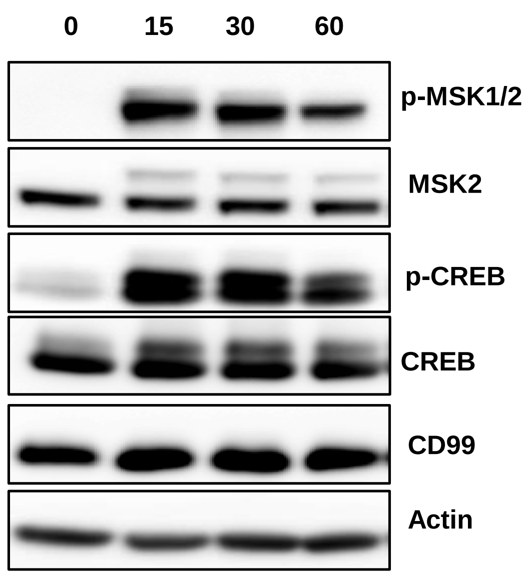

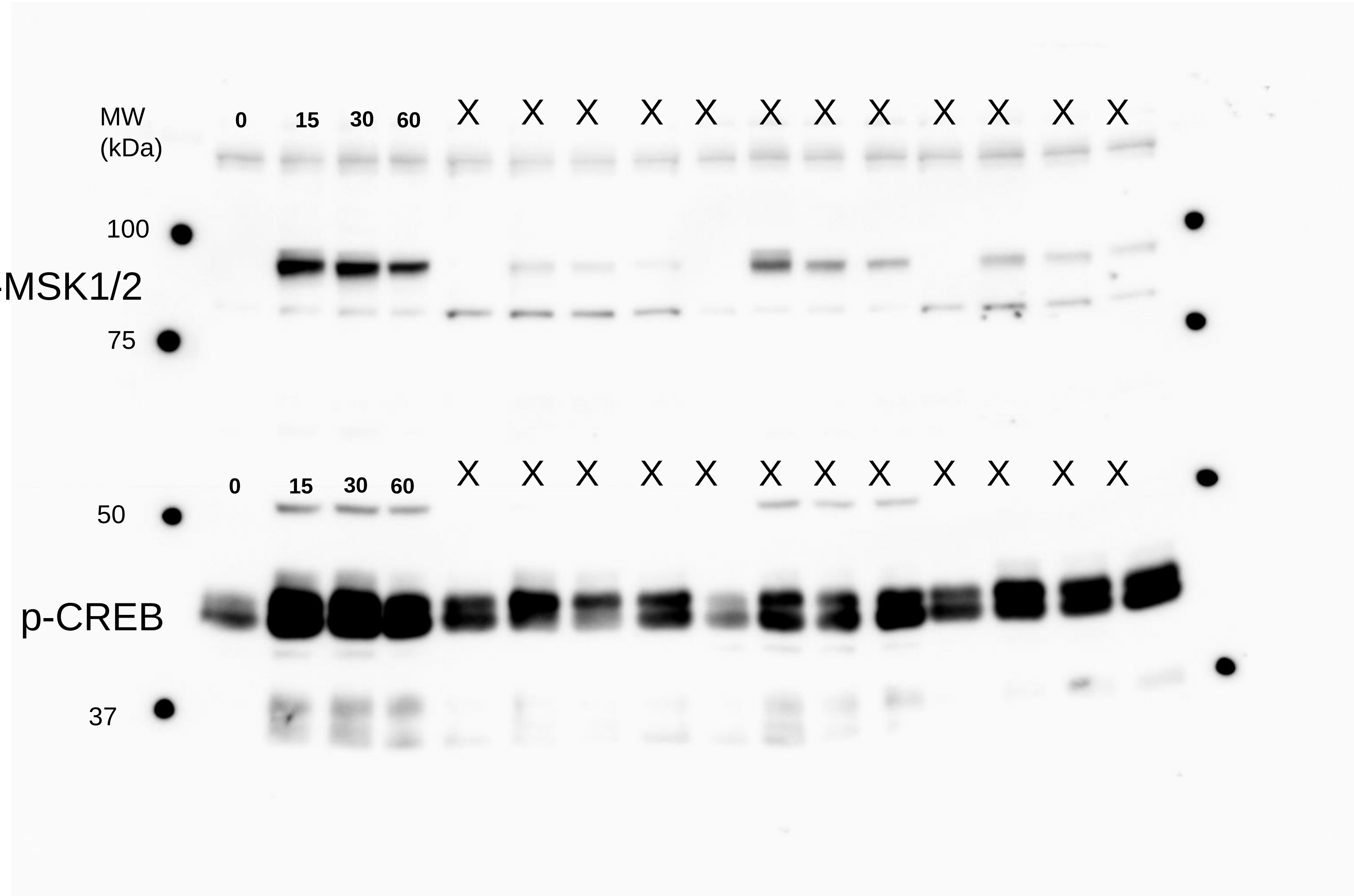

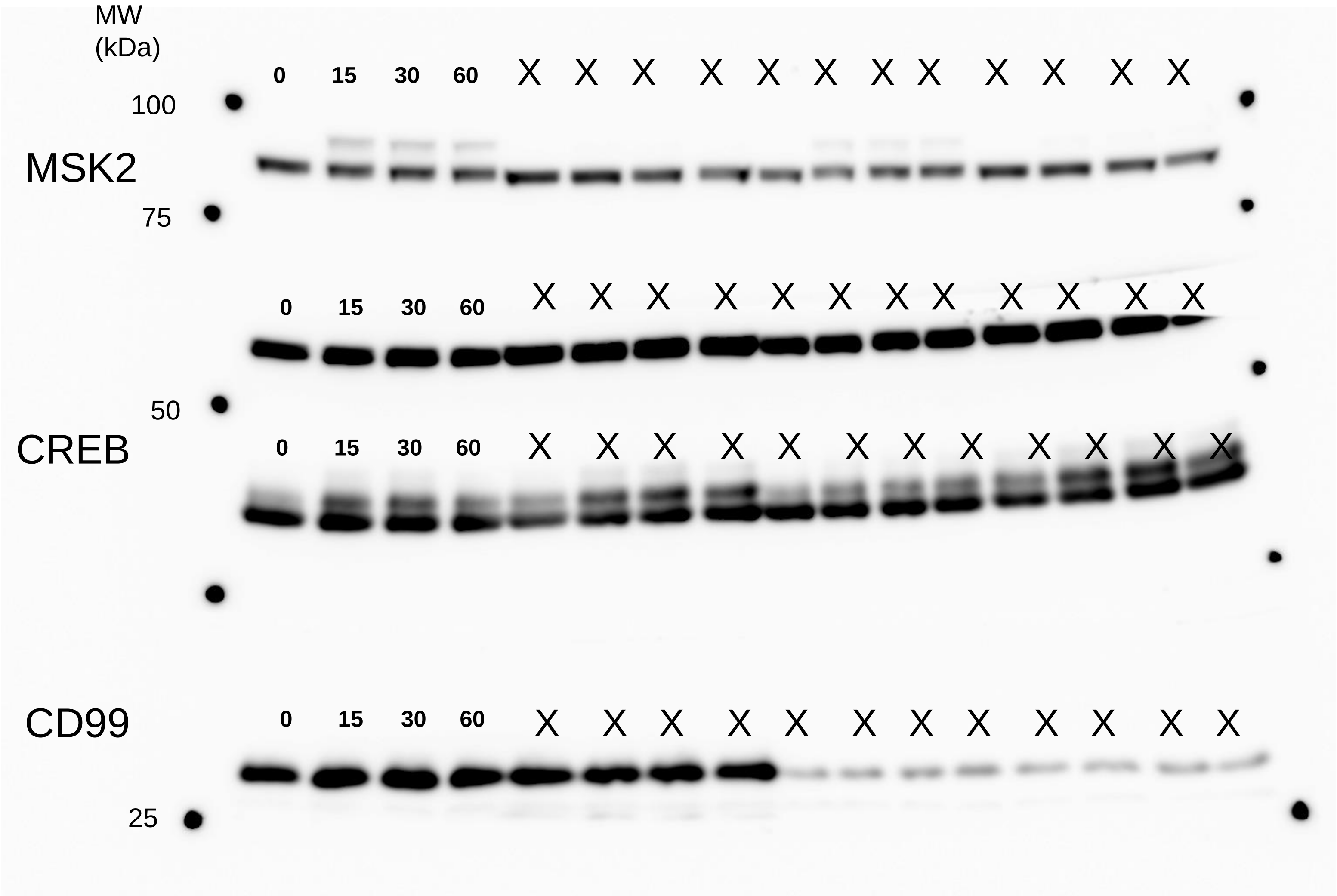

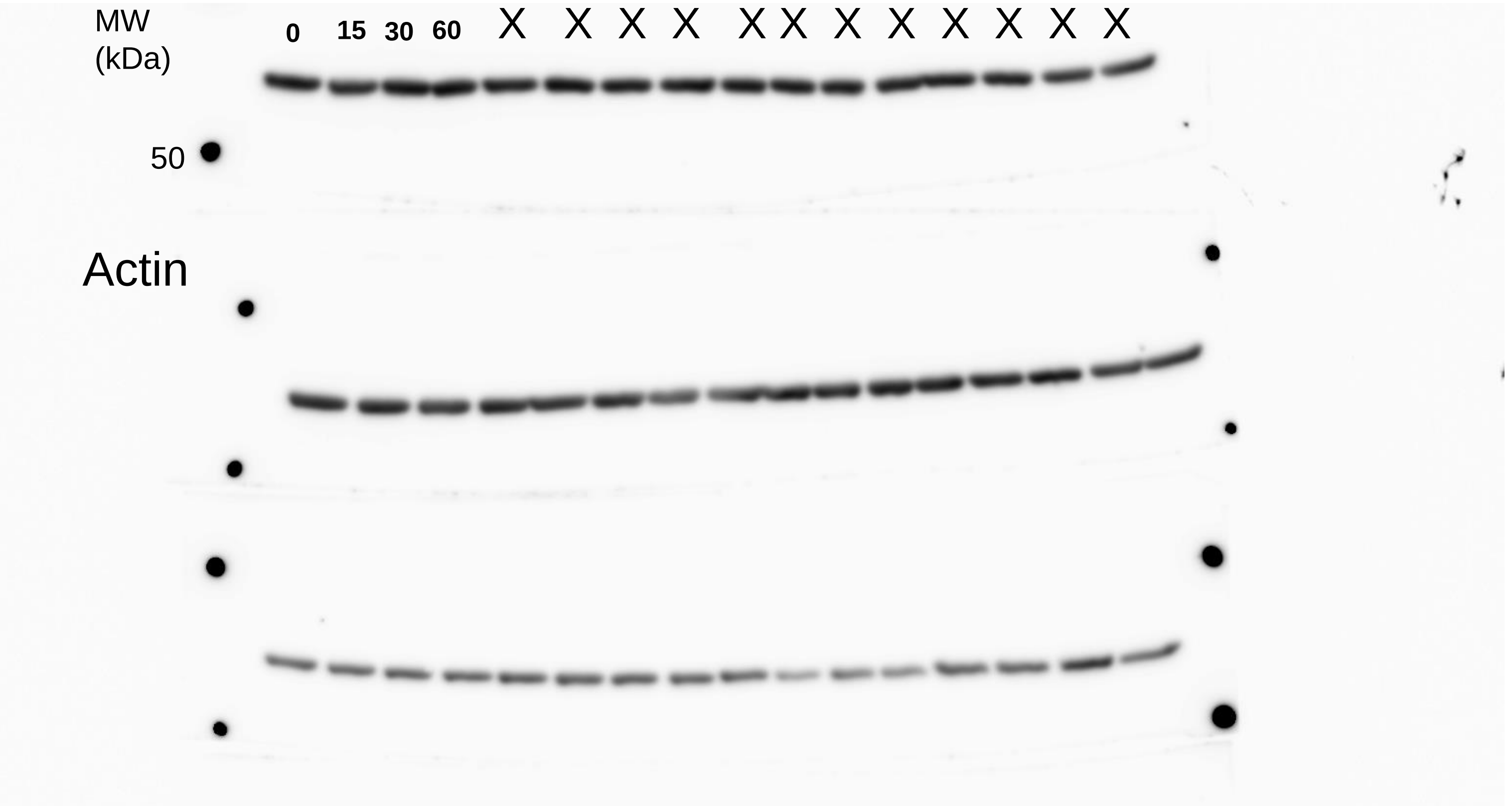

Sup Fıgure3C

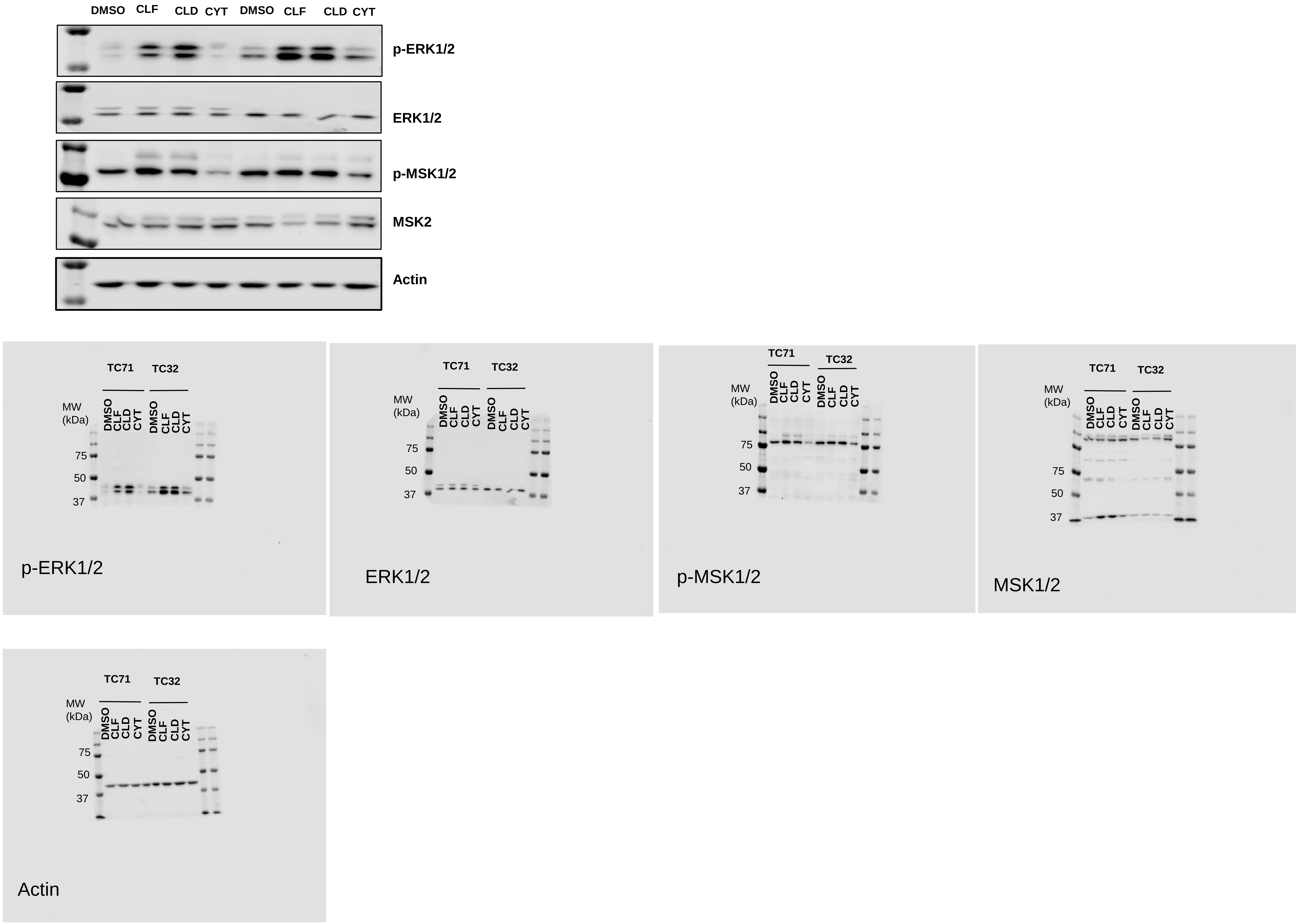

Figure S4

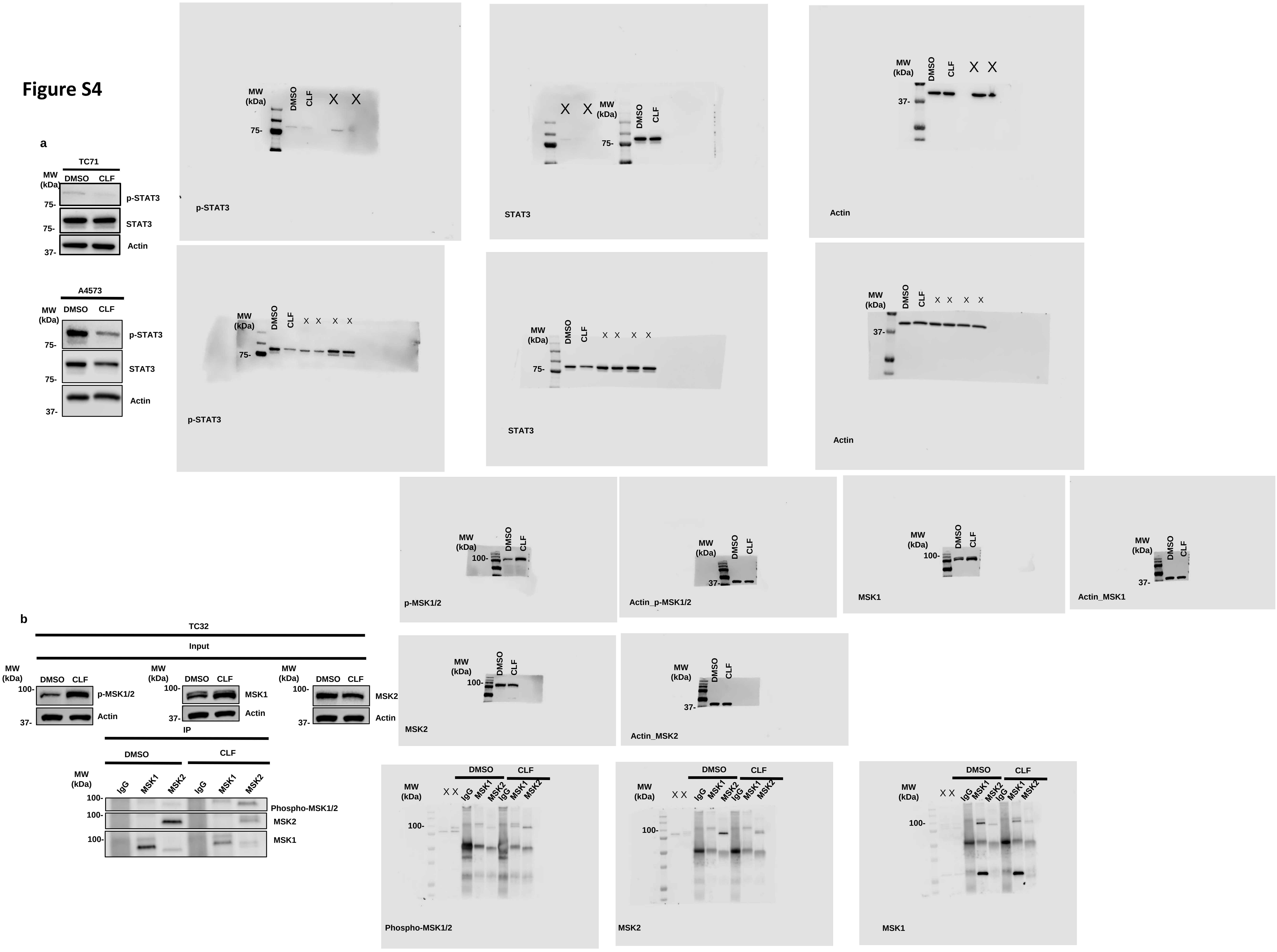

a
TC71
MW (kDa)
DMSO
CLF
p-STAT3
STAT3
Actin
75-
75-
37-
A4573
MW (kDa)
DMSO
CLF
p-STAT3
STAT3
Actin
75-
75-
37-
b
TC32
Input
MW (kDa)
DMSO
CLF
p-MSK1/2
Actin
MSK1
Actin
MSK2
Actin
100-
37-
IP
DMSO
CLF
IgG
MSK1
MSK2
IgG
MSK1
MSK2
Phospho-MSK1/2
MSK2
MSK1
100-
X
p-STAT3
STAT3
Actin
p-MSK1/2
Actin_p-MSK1/2
MSK1
Actin_MSK1
MSK2
Actin_MSK2
Phospho-MSK1/2
MSK2
MSK1

Figure S5A

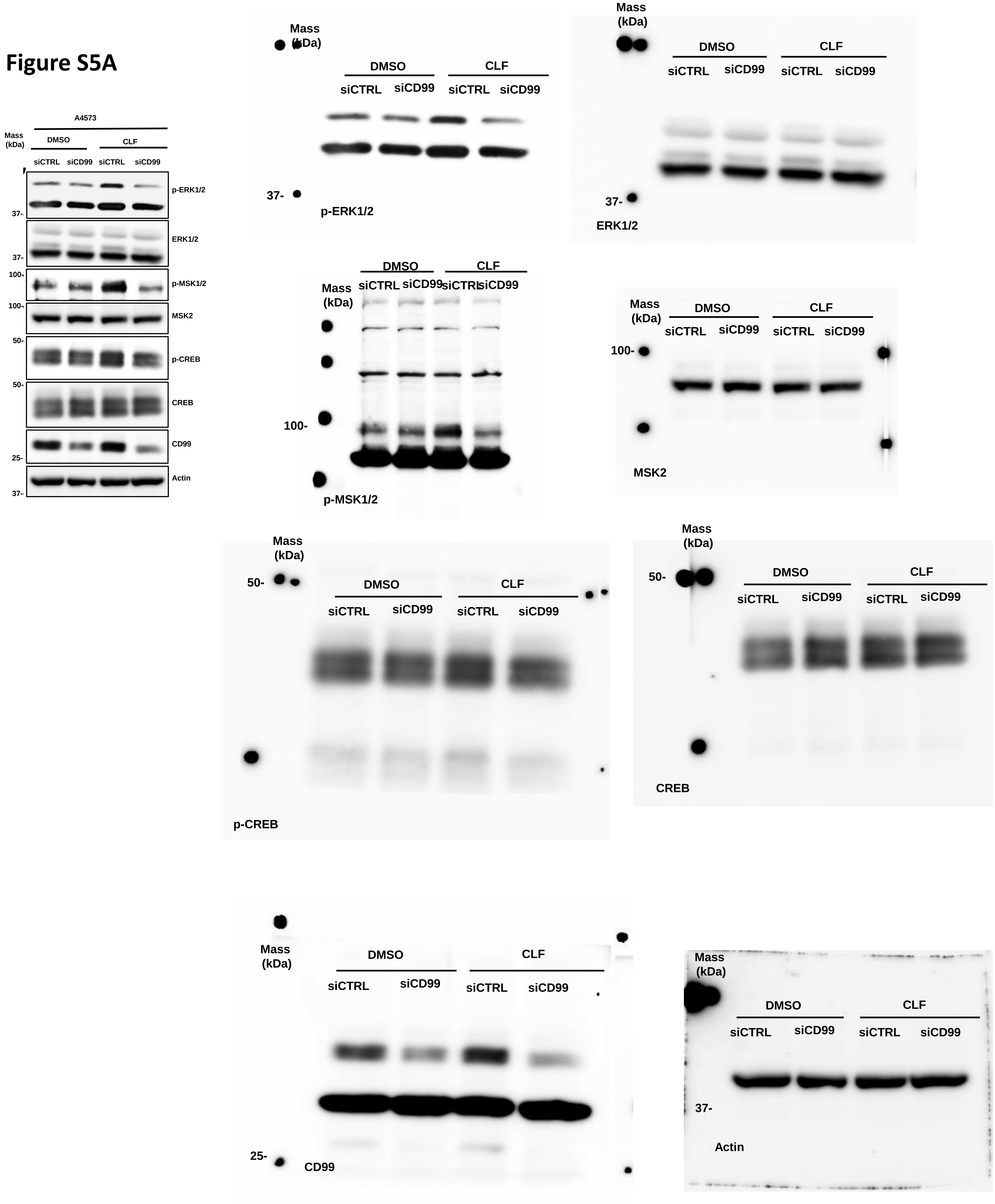

A4573
Mass (kDa)
DMSO
CLF
siCTRL
siCD99
p-ERK1/2
ERK1/2
p-MSK1/2
MSK2
p-CREB
CREB
CD99
Actin
37-
50-
100-
25-

Figure S5B

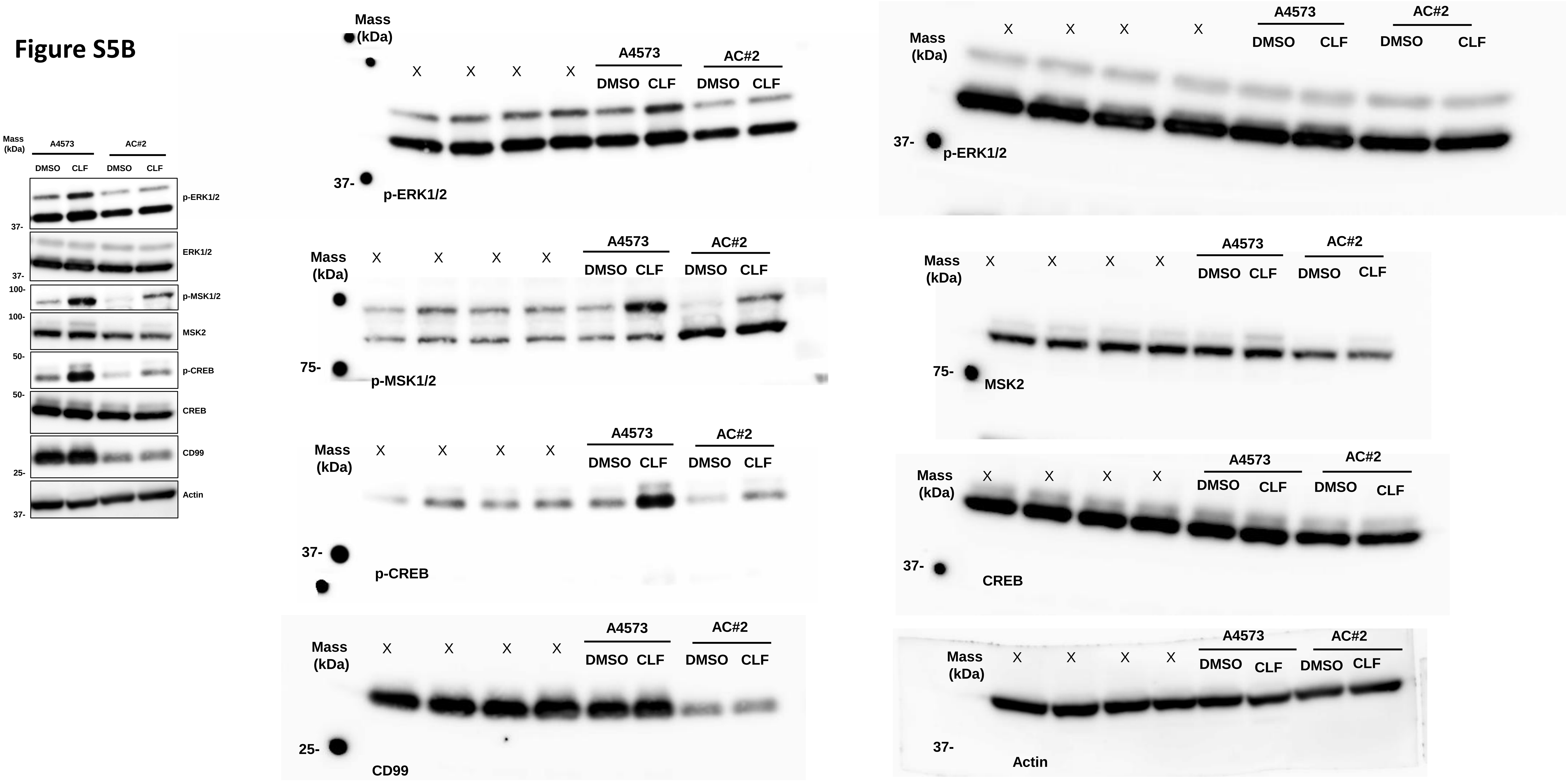

Mass (kDa)
A4573
AC#2
DMSO
CLF
DMSO
CLF
p-ERK1/2
ERK1/2
p-MSK1/2
MSK2
p-CREB
CREB
CD99
Actin
37-
100-
50-
25-
75-
X
